# Endothelial E-selectin inhibition improves acute myeloid leukaemia therapy by disrupting vascular niche-mediated chemoresistance

**DOI:** 10.1038/s41467-020-15817-5

**Published:** 2020-04-27

**Authors:** Valerie Barbier, Johanna Erbani, Corrine Fiveash, Julie M. Davies, Joshua Tay, Michael R. Tallack, Jessica Lowe, John L. Magnani, Diwakar R. Pattabiraman, Andrew C. Perkins, Jessica Lisle, John E. J. Rasko, Jean-Pierre Levesque, Ingrid G. Winkler

**Affiliations:** 1 Mater Research Institute—The University of Queensland, Translational Research Institute, Woolloongabba, QLD Australia; 2 GlycoMimetics Inc., Rockville, MD USA; 3 Molecular and Systems Biology, Norris Cotton Cancer Centre, Lebanon, NH USA; 4 Australian Centre for Blood Diseases, Monash University, Prahan, Vic Australia; 5 Gene and Stem Cell Therapy Program, Centenary Institute, University of Sydney, Sydney, NSW Australia; 6 Department of Cell and Molecular Therapies, Royal Prince Alfred Hospital, Camperdown, NSW Australia

**Keywords:** Cancer microenvironment, Cancer stem cells, Cancer therapy

## Abstract

The endothelial cell adhesion molecule E-selectin is a key component of the bone marrow hematopoietic stem cell (HSC) vascular niche regulating balance between HSC self-renewal and commitment. We now report in contrast, E-selectin directly triggers signaling pathways that promote malignant cell survival and regeneration. Using acute myeloid leukemia (AML) mouse models, we show AML blasts release inflammatory mediators that upregulate endothelial niche E-selectin expression. Alterations in cell-surface glycosylation associated with oncogenesis enhances AML blast binding to E-selectin and enable promotion of pro-survival signaling through AKT/NF-κB pathways. In vivo AML blasts with highest E-selectin binding potential are 12-fold more likely to survive chemotherapy and main contributors to disease relapse. Absence (in *Sele*^−/−^ hosts) or therapeutic blockade of E-selectin using small molecule mimetic GMI-1271/Uproleselan effectively inhibits this niche-mediated pro-survival signaling, dampens AML blast regeneration, and strongly synergizes with chemotherapy, doubling the duration of mouse survival over chemotherapy alone, whilst protecting endogenous HSC.

## Abstract (web summary)

The cell adhesion molecule E-selectin regulates haematopoietic stem cell self-renewal in the bone marrow vascular niche. Here, the authors show E-selectin adhesion directly induces survival signaling in acute myeloid leukaemia and therapeutic inhibition improves chemotherapy outcomes in mice.

## Introduction

Despite encouraging initial response, hematopoietic malignancies often relapse after therapy. Therapy resistance involves both cell-intrinsic and cell-extrinsic factors. Up to now most studies have focused on the cell intrinsic properties that enable therapy resistance^1^. Here, we describe a cell extrinsic niche-based pathway by which adhesion to vascular niche protein E-selectin directly mediates therapy-resistance, using acute myeloid leukemia (AML) as a model.

Malignant cells are known to have a greater survival advantage when lodged in peri-vascular locations^2,3^, however, the exact interactions mediating this survival advantage remain incompletely understood. The identification and targeting of such protective niche factors are critical to the development of novel strategies to eradicate residual resistant leukemia or cancer stem cells, to improve therapeutic outcomes.

In the bone marrow (BM), endothelial cells are known to form a vascular niche that plays a direct role in the support and regulation of hematopoietic stem cell (HSC) quiescence, self-renewal, activation, and homing^4,5^. We have previously found endothelial (E)-selectin to be a key vascular niche factor involved^6^.

Selectins are an evolutionarily ancient family of three cell adhesion molecules with a known role in mediating leukocyte rolling and homing to tissues. Two selectins are expressed on activated or inflamed endothelium: E-(endothelial)-selectin and P (platelet and endothelial)-selectin, often together with integrin ligands^7^. We have reported that adhesion to the endothelial cell-exclusive E-selectin (CD62E) is unique among these adhesion molecules in the regulation of the switch between dormancy and proliferation of HSCs^6^. When expressed on the BM endothelial niche, HSC contact with E-selectin directly triggers HSC activation and proliferation and induces commitment^6^. Conversely, absence or therapeutic blockade of E-selectin promotes HSC quiescence, boosting HSC self-renewal potential and chemo-resistance^6^. Herein we report that in the context of malignancy, E-selectin interactions instead play a direct role in promoting malignant cell survival.

Niche hijack describes how malignant cells manipulate stem cell niches to facilitate their own regeneration and/or survival, at the expense of nonmalignant host stem cells. To investigate vascular niche hijack during malignancy, we took advantage of preclinical mouse models of AML that mimic clinical features of 11q23-gene rearranged mono-myelocytic AML^8^. These models are generated by retroviral-transduction of murine HSC with human MLL-AF9 (11q23-rearranged) fusion oncogene which are then adoptively transferred into mice^8,9^.

We demonstrate that adhesion to a single vascular molecule initiates pro-survival signaling in AML blasts and that this niche-mediated therapy-resistance is dependent on receptor fucosylation in AML blasts, which is altered in response to oncogenic transformation^10^. We find absence (in *Sele*^−/−^ hosts), or therapeutic blockade of E-selectin (using small molecule antagonist GMI-1271/Uproleselan), at the same time as chemotherapy specifically sensitizes AML regenerating cells (LRC) and significantly improves overall treatment efficacy and mouse survival. These findings describe a potential role for the altered glycosylation, associated with oncogenesis^10^, and open a new strategy to significantly improve efficacy of therapy for AML and other malignancies by targeting a niche interaction (nichotherapy)^11^. Based on these data, clinical trials to determine the efficacy of E-selectin blockade (GMI-1271/Uproleselan) in combination with chemotherapy to treat adult AML are now in progress (NCT03701308 and NCT03616470).

## Results

### BM E-selectin expression increases during AML

**Fig. 1:**
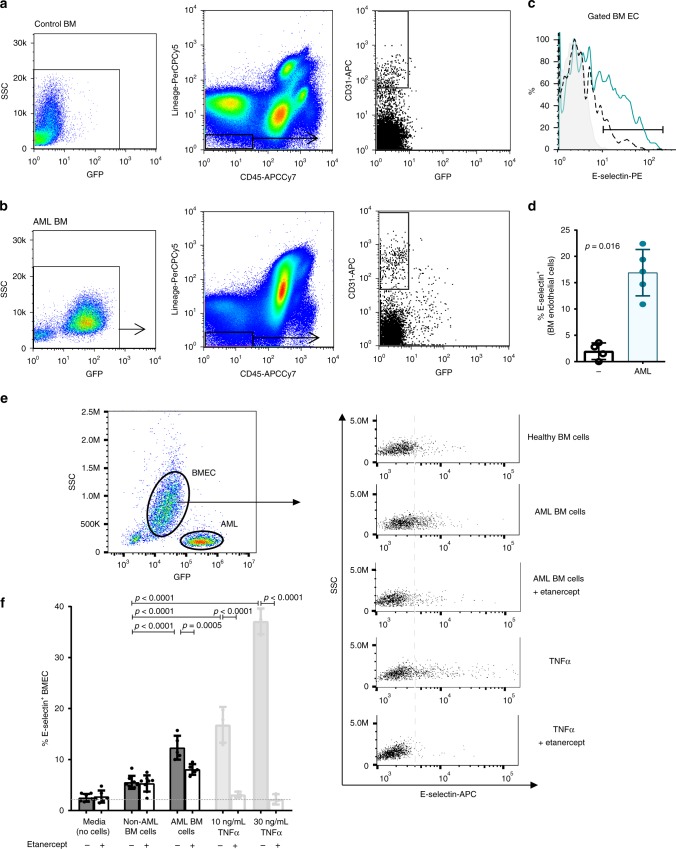
AML is associated with increased E-selectin expression on BM endothelial cells. **a**–**d** Endosteal BM was collected from mice with advanced GFP^+^ AML (MLL-AF9 induced, *n* = 5/group), or no leukemia controls (*n* = 4/group), stained for E-selectin cell surface expression on viable endothelial cells (7AAD^−^ lineage^−^CD45^−^ CD31^+^ GFP^−^) and analyzed by flow cytometry. **a**, **b** Gating strategy for BM endothelial cells from one representative mouse in each group: **a** Healthy non-leukemic control mouse, **b** leukemic mouse. **c** Overlay histogram comparing cell surface E-selectin expression on gated BM endothelial cells from mice with AML (blue line), non-leukemic mouse (black dashed line). Isotype negative control is gray filled. Bar shows E-selectin positive gate. **d** Histogram showing percent of endothelial cells that are positive for surface E-selectin expression. Each dot represents data from an individual mouse. Bars are mean ± S.D. Statistics: two-tailed *t* test. **e**, **f** BMEC-1 cells were cocultured with TNF-α (positive control for E-selectin activation), or with BM cells from healthy (non-leukemic) or leukemic mice ± TNF-α inhibitor etanercept for 16 h at 37 °C. Cocultured cells were then collected and stained for E-selectin expression on BMEC-1 cell surface and analyzed by flow cytometry. **e** Gating strategy for E-selectin expression on viable BMEC-1 cells. Shown are viable BMEC-1 gate (left) and surface E-selectin-APC expression (right). Representative dot plot from one well per group. **f** Histogram representing percentage of BMEC-1 expressing E-selectin after co-culture with medium alone, added BM cells from healthy and from leukemic AML mouse, or BMEC-1 with TNF-α, ± etanercept as indicated. Mean ± S.D. of pooled data from three independent experiments (*n* = 7,6,8,8,5,5,3,3,3,3 wells/group). Statistical significance calculated by one-way ANOVA with Bonferroni correction for multiple comparisons. Source data are provided as a Source Data file.

E-selectin is a vascular adhesion molecule expressed by endothelial cells following inflammation or injury^12^. We found cell surface E-selectin expression significantly increased (~eightfold) on BM endothelial cells in mice with AML compared to non-leukemic mice (Fig. 1a–d) and compared to AML in matched *Sele*^−/−^ hosts (Supplementary Fig. 1a–c). As endothelial E-selectin expression is induced by inflammatory cytokines via NF-κB response elements in the *Sele* gene promoter^12–14^, these data suggest AML generates inflammation in the BM which directly leads to increased E-selectin surface expression on endothelial cells. To confirm, fresh BM leukocytes from leukemic or healthy non-leukemic mice were cocultured in contact with BM endothelial cell line (BMEC-1) for 16 h, and expression of BMEC-1 cell surface E-selectin measured by flow cytometry. We found cocultures with BM cells from leukemic mice induced 2.5-fold higher E-selectin expression compared to cocultures with matched normal (non-leukemic) BM cells (Fig. 1e, f).

AML cells are reported to secrete inflammatory cytokines such as TNF-α^15,16^. TNF-α is known to directly induce E-selectin expression on endothelium^12–14^ (Fig. 1f). To confirm a role for AML-produced TNF-α in the upregulation of E-selectin, the effect of a TNF-α antagonist etanercept was tested in these co-cultures. We found addition of etanercept significantly reduced AML blast-mediated E-selectin expression on endothelial cells (by 41% ± 17%) over endothelial cell alone baseline (Fig. 1e, f). In contrast TNF-α blockade did not alter endothelial E-selectin expression levels in cocultures with non-leukemic BM cells. Together these data show AML produces inflammatory mediators such as TNF-α which activates endothelium resulting in increased E-selectin expression.

### AML blasts show increased E-selectin-binding potential

A variety of cell surface glycoproteins and glycolipids may act as E-selectin receptors when appropriately glycosylated to form the sialyl Lewis^x/a^ (sLe^x/a^) carbohydrate structure. For E-selectin binding, putative receptors must be post-transcriptionally regulated via a variety of glycosyl transferases^17,18^, including α(1,3)-fucosylation of a terminal N-acetyl-d-glucosamine sugar by α(1,3) fucosyl-transferases (FUT), usually FUT7 and/or FUT4 (as well as FUT9 in humans)^19,20^. One of the hallmarks of malignant transformation is the alterations in cell surface glycosylation, including fucosylation, which often results in a de novo acquisition of E-selectin-binding potential^21^. De novo, or increased, expression of the FUTs by tumor cells is associated with poorer clinical outcomes^22–27^.

**Fig. 2:**
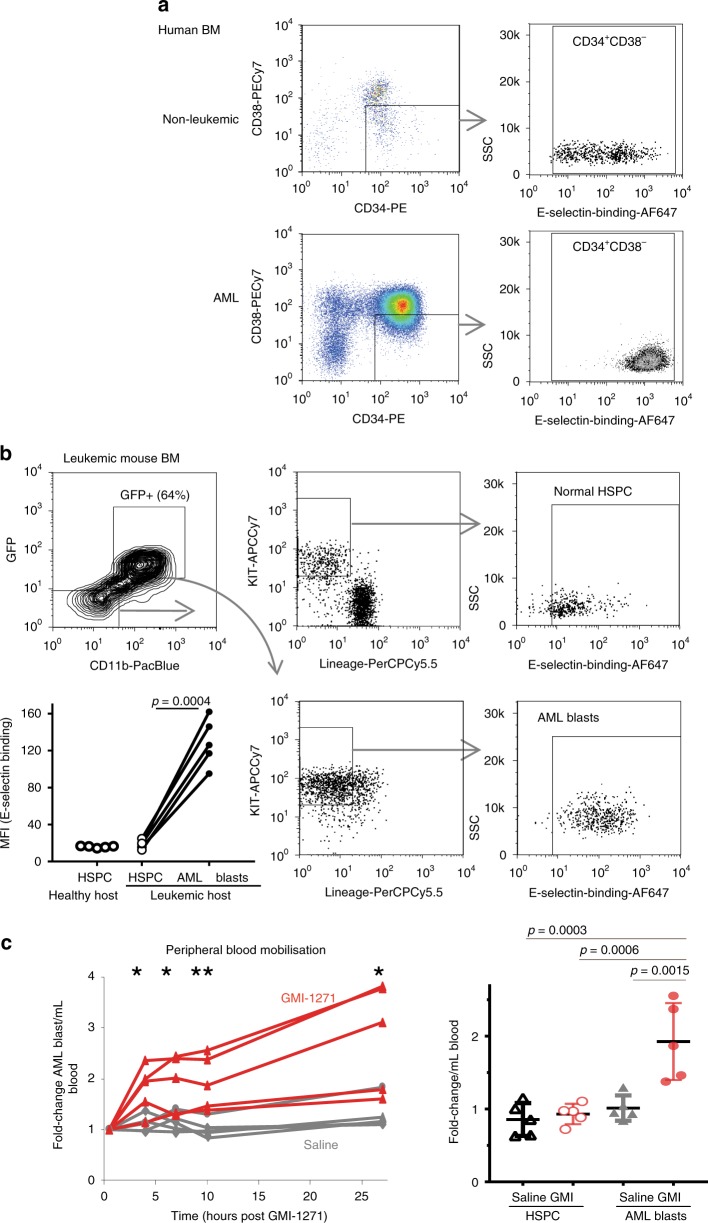
E-selectin binding-potential is increased in AML blasts and plays a role in BM retention. **a** Representative Flow cytometry gating strategy for healthy lineage^−^ CD34^+^ CD38^−^ cells (*n* = 2) compared to patient CD34^+^ CD38^−^ AML blasts (*n* = 2) on left, and their E-selectin-binding potential after labell with fluorescent recombinant human E-selectin–IgM on right, **b** Left panel: Gating strategy for Murine BM HSPC (GFP^−^ Lineage^−^ CD11b^−^ KIT^+^) and MLL-AF9 AML blasts (GFP^+^ Lineage^neg^ CD11b^+^ KIT^+^) with E-selectin-IgM binding potential (Right panel). Graph shows difference in median florescence intensity (MFI) of E-selectin binding in HSPC from healthy (non-leukemic) hosts, and between non-malignant HSPC and AML blasts within same host. Each linked pair of dots are data from same mouse (*n* = 5/group, two-tailed paired *t* test *p* = 0.0004). **c** Bolus administration of E-selectin antagonist GMI-1271 in vivo selectively mobilizes a small proportion of AML blasts into the peripheral blood. Left panel; time-course of relative change in GFP^+^ KIT^+^ AML blasts circulating in peripheral blood post GMI-1271 (red lines) or saline vehicle injection (gray lines). Each line represents data from an individual mouse (*n* = 5/group). Expressed as fold above starting (zero time) baseline. Statistics: two-tailed *t* test; 4 h *p* = 0.0153; 7 h *p* = 0.0230; 10 h *p* = 0.0063; 27 h *p* = 0.0153. Right panel; HSPC (GFP^neg^ Lineage^neg^ CD11b^−^ KIT^+^) or AML blasts (GFP^+^Lineage^neg^ CD11b^+^ KIT^+^) in peripheral blood. Shown is fold-change at 10 h post injection of GMI-1271 (GMI, red circles) or saline vehicle (gray triangles) above baseline. Each dot represents data from an individual mouse. Mean ± S.D. (*n* = 5 mice/group). One way-ANOVA multiple comparisons with post hoc Bonferroni correction. Source data are provided as a Source Data file.

Using fluorescently labeled recombinant human E-selectin–IgM fusion protein^6^, we found that healthy human CD34^+^ CD38^−^ HSC display a range of E-selectin binding potentials, whereas primary AML CD34^+^ CD38^−^ blasts bind strongly (Fig. 2a). In mice, E-selectin binding was similarly heterogeneous on normal lineage-negative CD11b^−^ KIT^+^ hematopoietic stem and progenitor cells (HSPC) but strongly increased on GFP^+^ KIT^+^ MLL-AF9 AML blasts (Fig. 2b). To confirm the importance of fucosyl transferases FUT4 and FUT7 in this adhesive interaction, AML was generated from *Fut4*/*Fut7* double gene-deleted mice. We found complete abrogation of E-selectin-binding-potential when both *Fut4* and *Fut7* were absent (Supplementary Fig. 2), confirming an absolute requirement of cell surface fucosylation for E-selectin binding.

Next the potential functional role for the increased E-selectin binding observed on AML blasts was investigated by administration of a single bolus dose of selective small-molecule E-selectin antagonist GMI-1271 to leukemic mice. GMI-1271/Uproleselan is highly specific for E-selectin with IC_50_ of 2.4 µM for E-selectin-binding compared to >10,000 µM for related P-selectin, 4516 µM for L-selectin and is function-blocking at plasma concentration of 38 µM in patients.

We found administration of GMI-1271 to leukemic mice induced some mobilization of leukemic blasts into peripheral blood to levels three to fourfold above starting levels, that persisted for 24 h when compared to saline injected leukemic controls (Fig. 2c, left). No similar mobilization of non-leukemic endogenous HSPCs was observed following GMI-1271 administration (Fig. 2c, right). These data suggest that E-selectin-mediated interactions may play some role in the retention of AML blasts within the BM niche, but not in HSPC retention. Despite this, when compared to the total number of AML blasts in the BM and spleen of a leukemic mouse, only ~1% of total body AML blast relocated to the peripheral blood following GMI-1271 administration, or in a *Sele*^−/−^ host (Supplementary Fig. 3). These data suggest that the mobilization of AML blasts in blood following bolus GMI-1271 administration (observed in Fig. 2c) is minor compared to total body (BM/spleen) AML blast numbers, or to the overall variation between mice, and unlikely to significantly impact overall AML therapy response.

### Blockade of E-selectin sensitizes AML LRC to chemotherapy

**Fig. 3:**
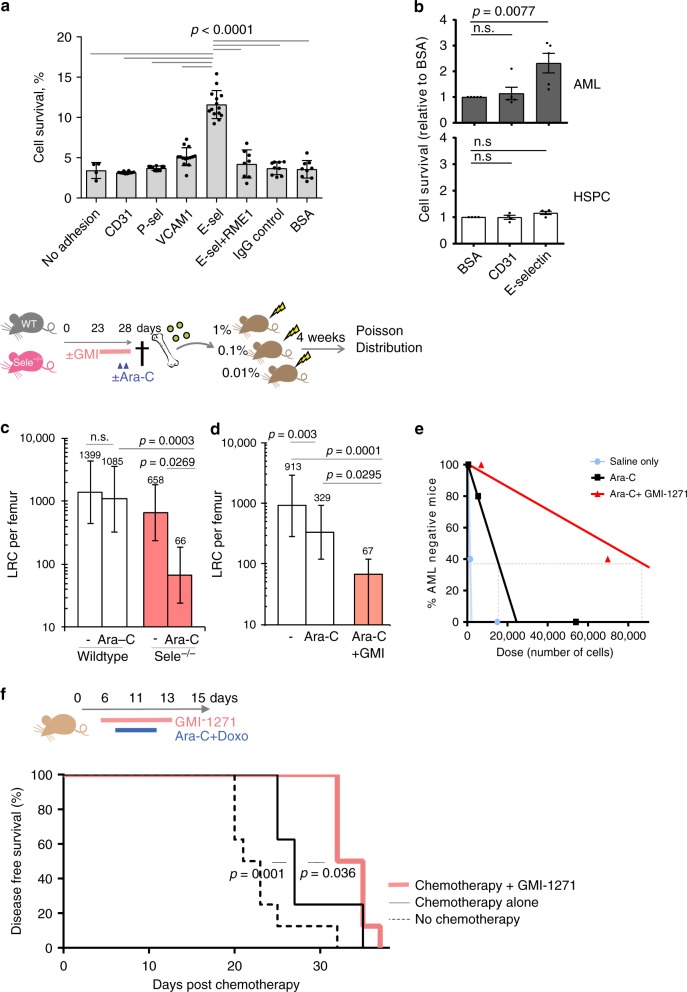
Absence or therapeutic blockade of E-selectin sensitizes AML LRCs to chemotherapy and extends duration of disease-free survival in mice. **a**, **b** AML cells or non-leukemic Kit^+^ HSPC from mouse BM were cultured for 3 days with Ara-C in wells precoated with adhesion molecules as indicated. In some wells, E-selectin blocking antibody RME-1 or isotype control added. **a** Plotted are percentage cell survival compared to matched non-chemotherapy-treated wells. Mean ± SD of pooled data from two experiments (*n* = 4,10,10,14,14,8,9,9 wells/condition). Statistics: one-way ANOVA multiple comparisons Bonferroni correction. **b** Histogram showing survival of Kit^+^ AML cells (top panel) compared to healthy (non-leukemic) HSPC (bottom panel) after Ara-C treatment in vitro. Shown as fold-change compared to control (BSA) wells. Mean ± SEM of pooled data from 4 (HSPC) or 5 (AML blasts) independent experiments (*n* = 5 wells/condition/experiment). Statistics: one-way ANOVA multiple comparisons Bonferroni correction. **c**–**e** Experimental outline. Mice with advanced MLL-AF9 AML were administered ±GMI-1271 40 mg kg^−1^ BiD, for 5 days prior to and during 24 h chemotherapy (900 mg kg^−1^ BiD Ara-C) in wildtype or *Sele*^−/−^ hosts as indicated. The number of surviving leukemia repopulating cells (LRC) then measured by serial-dilution transplantation of 1%, 0.1%, 0.01% total BM from one femur (*n* = 5 donors, *n* = 5 recipients per cell dose). Surviving number of LRC per femur and statistical comparison analysis calculated by Poisson distribution statistics using L-calc software. Shown is calculated LRC per femur ±95% CI. Degrees Freedom 2. **c** LRC per femur in wildtype versus *Sele*^−/−^ hosts ±95%CI. **d** LRC per femur in saline vs. GMI-1271-treated mice ±95%CI. **e** Frequency regression of AML-negative recipients from (**d**) as function of transplanted BM cell number. Vehicle injected (black line), GMI-1271-treated mice (red line). Non-chemotherapy treated-mice (blue line). Horizontal dotted gray line is 37.5% threshold used for determining LRC frequency. **f** Wildtype mice with AML were administered GMI-1271 40 mg kg^−1^ BiD or saline before and during 5-day induction chemotherapy regimen (doxorubicin 1 mg kg^−1^ 3 d and Ara-C 100 mg kg^−1^ 5 d) and monitored for duration of disease-free survival. Shown is Kaplan Meier curve (*n* = 8 mice/group). Statistics: Log Rank (Mandel–Cox) survival curve comparison. Source data are provided as a Source Data file.

To investigate if E-selectin-mediated adhesion directly promotes therapy-resistance and survival, AML blasts were adhered to immobilized recombinant E- and P-selectin-human IgG1-Fc proteins, as well as to a range of other recombinant adhesion molecules involved in leukocyte homing. Other adhesion molecules tested, include CD31 (platelet endothelial cell adhesion molecule, PECAM-1) and CD106 (vascular cell adhesion molecule 1, VCAM-1). We found that adhesion to E-selectin was unique in promoting 4.6-fold (*p* < 0.001) greater survival of AML blasts to cytarabine treatment in vitro (Fig. 3a). This enhanced chemoresistance was restricted to E-selectin adhesion only and not mediated by adhesion to any of the other leukocyte homing molecules tested, and also specific as it could be inhibited by addition of anti-E-selectin blocking monoclonal antibody RME-1 (Fig. 3a). Similar E-selectin-specific mediated chemoresistance was also observed using human CD34^+^ AML cell line KG1a (shown later), indicating that this endothelial niche-mediated pro-survival signaling pathway is common between human and mouse AML. In contrast, no similar E-selectin-mediated chemo-resistance was observed in normal BM KIT^+^ HSPC harvested from non-leukemic mice (Fig. 3b) confirming malignant AML blasts respond differently to E-selectin-adhesion compared to BM HPSC.

AML has a similar hierarchical structure to normal HSC, with only a small proportion of leukemic blasts (known as LRC) endowed with the functional capacity to regenerate leukemia in vivo^28,29^. It is only if these rare LRC survive chemotherapy that disease relapses^30,31^. To investigate whether functional LRC were among those protected by E-selectin-mediated interactions at the BM vascular niche, we developed a quantitative in vivo LRC chemo-sensitivity assay based on the principle of limiting-dilution transplantations, similar to the gold standard assays for enumerating functional HSCs^32^. Cohorts of E-selectin gene-deleted (*Sele*^−*/*−^) or GMI-1271-treated, wild-type mice with advanced AML were administered high-dose cytarabine (Ara-C) or vehicle control. At 24 h post cytarabine, femoral BM cells were collected and transplanted into cohorts of wild-type recipient mice in limiting dilutions (1%, 0.1%, or 0.01% femoral content). The proportions of recipients that themselves developed leukemia by 4 weeks were then used to calculate frequency and number of chemotherapy-surviving LRCs using Poisson’s distribution statistics (Fig. 3c–e). These studies confirmed that although cytarabine administration reduces total AML burden, (Supplementary Fig. 4) virtually all LRC survive and re-establish the disease (Fig. 3c). However, when E-selectin was absent (in *Sele*^−/−^ hosts, Fig. 3c) or therapeutically blocked with GMI-1271, (Fig. 3d, e) a four to tenfold reduction of LRC survival was observed. Together these data confirm that E-selectin alone provides significant niche-mediated pro-survival signaling for LRC in vivo.

To determine whether therapeutic E-selectin niche blockade synergized with chemotherapy to boost overall disease-free survival, leukemic mice were administered E-selectin antagonist together with a standard induction chemotherapy regimen (doxorubicin/cytarabine) then monitored for survival (Fig. 3f). Although induction chemotherapy alone significantly enhanced median disease-free survival of mice by 23% over no chemotherapy control (from 22 to 27 days), this further doubled when E-selectin antagonist was co-administered (to median 34 days). Together these data indicate that therapeutically blocking this vascular niche-mediated survival pathway strongly synergizes with conventional AML treatment regimens to improve therapeutic efficacy and extend overall survival.

**Fig. 4:**
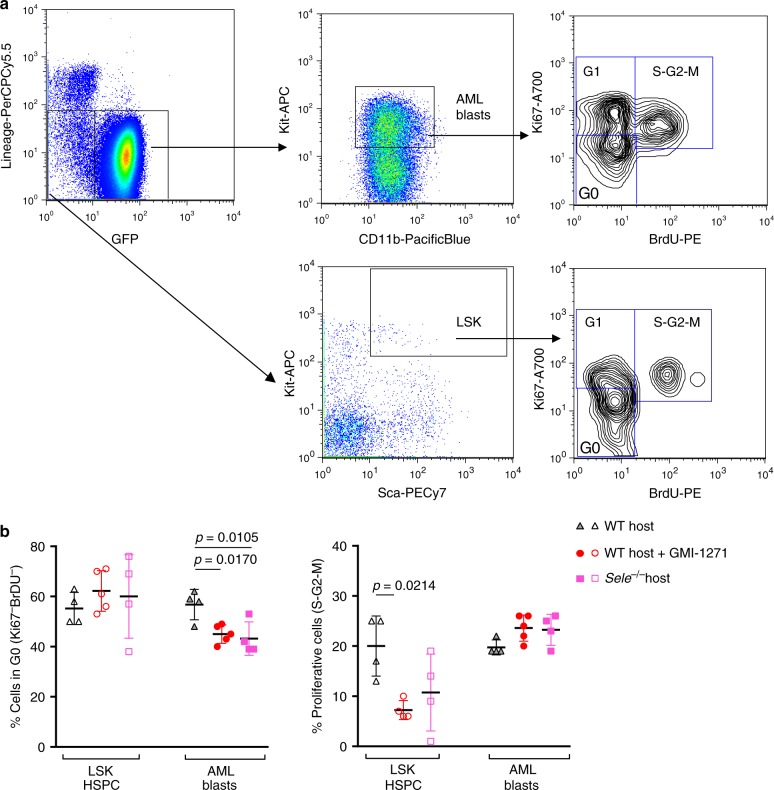
Absence or blockade of E-selectin stimulates quiescent AML blasts to cycle, while HSPC remain dormant. **a** Flow cytometry dot plots showing gating strategy for AML blasts (GFP^+^ Lin^−^KIT^+^CD11b^+^) and HSPC (GFP^−^ Lin^−^ KIT^+^ Sca1^+^, LSK) within the same leukemic host from mice administered 24 h BrdU. The cell cycle phases were defined as G_0_ (Ki-67^−^, BrdU^−^), G_1_ (Ki-67^+^, BrdU^−^) and S-G_2_-M (Ki-67^+^, BrdU^+^). **b** Histograms show percentage of bone marrow LSK HSPC and AML blasts in quiescence (phase G_0_, left panel*)* and proliferative (BrdU^+^, right panel). Each dot represents data from an individual mouse. Shown are mean ± S.D., *n* = 4,5,4 mice/group. Statistical analysis performed by one-way ANOVA multiple comparison Bonferroni correction. Source data are provided as a Source Data file.

To further investigate the differences between HSPC and AML response to E-selectin blockade, we investigated how loss of E-selectin binding affects cycling of non-malignant HSPC cells (GFP^−^ lineage^−^ Sca-1^+^ KIT^+^) compared to AML blasts (GFP^+^ lineage^−^ CD11b^+^ KIT^+^) within the same host. To this aim, cell cycle analysis was performed by BrdU incorporation and Ki-67 staining in cohorts of E-selectin gene-deleted (*Sele*^−*/*−^) or wild-type host mice ±GMI-1271 administration (Fig. 4a, b). This analysis confirmed that absence or blockade of E-selectin reduced the proportion of quiescent AML blasts (in G_0_ phase of cell cycle) by ~25% compared to saline-vehicle injected wildtype host controls (Fig. 4b, left panel). In regards to proliferation measured by 24 h 5-bromo-2′-deoxyuridine (BrdU) incorporation in vivo, blockade of E-selectin significantly dampened host LSK HSPC proliferation by ~50% (*p* = 0.0214), with a similar trend in *Sele*^−*/*−^ mice and consistent with our prior findings^6^. In contrast AML blasts within the same host showed no changes in, or a slight trend toward increased proliferation (Fig. 4b, right panel). Altogether, these data demonstrate the outcomes of E-selectin blockade to be different for LSK HSPC (dampens cycling) compared to AML with increased cell cycling (Fig. 4b) and chemo-sensitivity (Fig. 3c–f).

### E-selectin-binding associates with chemo-survival in AML

**Fig. 5:**
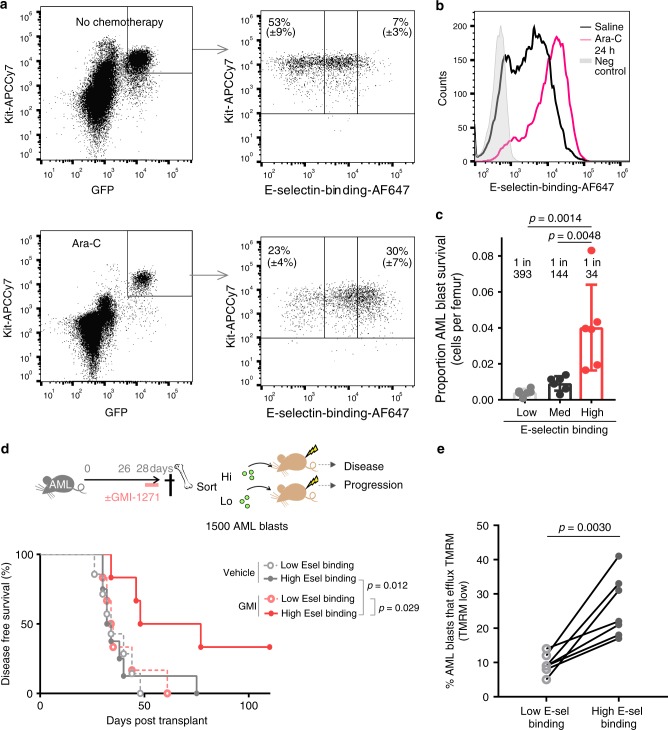
High E-selectin-binding potential characterizes the AML blasts more likely to survive chemotherapy. **a**–**c** Mice with MLL-AF9 leukemia (*n* = 6/group) were administered 24 h high-dose cytarabine (Ara-C) chemotherapy, or vehicle control, prior to harvest of BM cells for flow cytometry analyses. **a** Dot plots show gating strategy and altered distribution of E-selectin-binding potential among BM AML blasts (GFP^+^ KIT^+^) before (top panel) and after chemotherapy (bottom panel). Representative dot plot from one mouse per group. **b** Fluorescence histogram overlay showing E-selectin-binding intensity of BM AML blasts before (black line) and after (pink line) chemotherapy. Grey fill is no binding negative control (*n* = 6 mice/group). Representative mouse shown. **c** Percentage of surviving phenotypic AML blasts after 24 h cytarabine administration compared to starting numbers per femur for each E-selectin-binding category (low, medium (med), high gates, as defined in dot plot panel in (5a) above. Mean ± SD of *n* = 6 mice/group. Statistical analysis, one-way ANOVA multiple comparison Bonferroni correction. **d** Experimental outline, (*n* = 6) mice were separated by FACS and 1500 sorted AML blasts (GFP^+^ KIT^+^) with highest or lowest E-selectin-binding (using the gating strategy shown in top panel above) were transplanted into 2.5 Gy conditioned recipient mice (*n* = 8/recipients group). In one cohort of donors GMI-1271 was administered 200 mg kg^−1^ BiD for last 48 h prior to harvest and sort. (Bottom) Kaplan Meier disease-free survival of recipient mice transplanted with 1500 sorted AML blasts. Solid lines represent survival of recipients of high E-selectin-binding whereas dashed lines are recipients of low E-selectin-binding AML blasts. Grey curves indicate recipients of AML blasts from vehicle treated donors, while red/orange indicates recipients of AML blasts from GMI-1271-treated donors. Statistics, Log-Rank (Mantel–Cox) survival curve comparison. **e** Percentage of AML blasts able to efflux Rhodamine-derivative TMRM dye by flow cytometry, within lowest or highest E-selectin binding groups (gating strategy shown in Supplementary Fig. 5). Each pair of dots are data from same individual mouse (*n* = 7 mice/group). Statistical significance calculated by two-tailed paired *t* test. Source data are provided as a Source Data file.

We next investigated the role of E-selectin-binding potential in mediating AML therapy relapse. Following chemotherapy administration, we observed a pronounced enrichment of AML blasts with higher E-selectin binding potential in the BM (Fig. 5a–c). Specifically, the brightest 7% of AML blasts for E-selectin-binding-potential were >12-fold more likely to survive post-chemotherapy in vivo compared to lowest 50% for E-selectin binding (Fig. 5c).

To determine whether high E-selectin-binding potential was a prospective marker of LRCs, AML blasts from murine BM were sorted based on E-selectin-binding potential (highest or lowest) and transplanted into recipients (at exactly 1500 AML blasts per recipient) (Fig. 5d). Analysis of the time to relapse in these recipient mice (Fig. 5d) suggests no significant intrinsic difference in regenerative potential between sorted AML blasts with highest or lowest E-selectin binding potential (compare grey lines). However, when E-selectin antagonist was administered for the last 48 h prior to BM harvest, median survival duration doubled in the recipients of high E-selectin-binding AML cells from 33 to 62.5 days (*p* = 0.012) (Fig. 5d, grey vs. red solid lines) indicating the blockade of E-selectin interactions significantly dampened LRC potential to initiate “relapse” under these conditions.

To determine whether E-selectin-binding potential correlates with other characteristics connected with intrinsic drug resistance in AML, we investigated cell potential to efflux rhodamine fluorescent dye (Tetramethylrhodamine, methyl ester; TMRM). Higher efflux potential is associated with greater intrinsic capacity to efflux (export) drugs^33^ and increased therapy-resistance^34^. We found that AML blasts with greatest rhodamine efflux were wholly contained within the highest E-selectin-binding cells (Fig. 5e, Supplementary Fig. 5). Overall, a 2.5-fold (*p* = 0.0030) increase in functional efflux potential was observed in AML blasts with high E-selectin-binding potential, compared to lower binding AML when corrected against same stain in presence of drug efflux blocker verapamil as “no efflux” control (Fig. 5e, Supplementary Fig. 5). Together these data suggest that high E-selectin-binding potential and greater intrinsic drug export potential correlate in AML blasts in our model. Interestingly, non-malignant LSK HSPC in the same host demonstrated overall higher potential to efflux TMRM compared to AML blasts, and efflux potential in HSPC did not correlate with E-selectin binding (Supplementary Fig. 5). These studies again demonstrate difference between HSPC and AML blasts in regards to cell surface glycosylation.

### E-selectin activates pro-survival signaling in AML

**Fig. 6:**
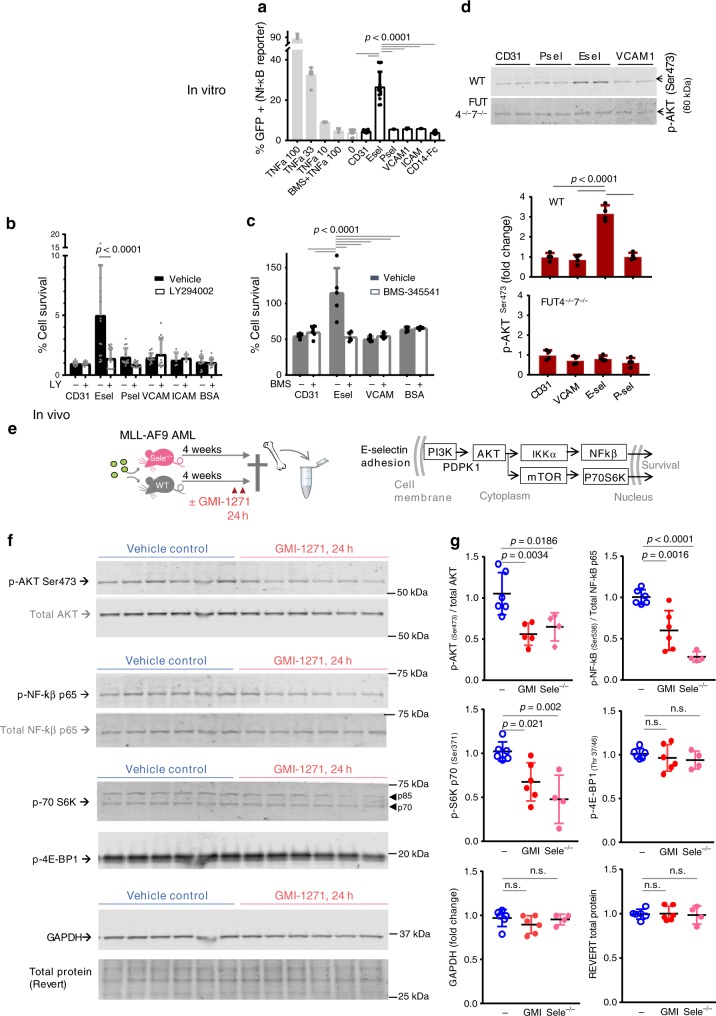
E-selectin adhesion activates AML pro-survival NF-κB signaling in vitro whereas E-selectin blockade in vivo dampens AKT/NF-κB signaling. All analyses one-way ANOVA multiple comparison Bonferroni correction. All plots mean ± SD. **a**–**c** In vitro studies. **a** NF-κB GFP reporter Raw264.7 cell line cultured in wells pre-coated with mouse adhesion molecules ± TNF-α as indicated (concentrations in ng mL^−1^). Intracellular activation of NF-κB-mediated transcription after 3 h adhesion measured by flow cytometry as percentage of viable cells. Pooled data from three independent experiments (*n* = 3,3,5 wells/group/experiment). **b**, **c** E-selectin adhesion-mediated chemoresistance is abrogated by small synthetic PI3K (LY294002) or NF-κB (BMS-345541) inhibitors. AML blasts were seeded into pre-coated wells for 3 days ± cytarabine. Data show percentage of surviving AML cells. **b** Murine AML blasts cultured with 25 ng mL^−1^ cytarabine ± 2.5 µM LY294002 (*n* = 5 wells/condition; 3 independent experiments). **c** Human CD34^+^ AML cell line KG1a cultured in the presence of 10 μg mL^−1^ cytarabine ± 10 µM BMS-345541 (*n* = 5 wells/condition). **d** p-AKT^Ser473^ phosphorylation in MLL-AF9 AML blasts generated from wildtype or *Fut4*^−*/*−^*Fut7*^−*/*−^ double gene-deleted BM cells, were seeded in pre-coated wells (25 min 37 °C) then lysed for quantitative immunoblotting (Li-Cor). Cell lysate from 30,000 adherent AML blasts from WT (upper blot) and from *Fut4*^−*/*−^*Fut7*^−/−^ AML KIT^+^ GFP^+^ blasts (lower blot) loaded per lane. Histogram shows fold increase in p-AKT^Ser473^ after total loaded protein normalization. Data pooled from two independent experiments, *n* = 2 wells/experiment. **e**–**g** In vivo studies. **e** Outline of experimental plan and intracellular signaling pathways investigated. **f** Quantitative immunoblotting of BM lysates with indicated antibodies using linear Li-COR detection. Lysates were from wildtype leukemia mice administered GMI-1271 (24 h 200 mg kg^−1^, BiD) or vehicle control. Each lane contains 15 µg BM lysate protein from an individual mouse (*n* = 6 mice/group). **g** Quantitative band intensity analyses by Li-COR from blots in (6f, *n* = 6/group). Includes additional data from same experiment with AML transplanted into (*n* = 4) *Sele*^−/−^ hosts loaded on parallel blot (Supplementary Fig. 6). Each dot represents data from a single mouse expressed as fold-change over average for saline vehicle injected controls. Source data (including full original scans of all blots) are provided as a Source Data file.

Chemo-resistance in malignant cells is frequently mediated by intracellular signaling through PI3K/AKT/NF-κB or mTOR pathways^35^. To determine whether E-selectin-mediated adhesion directly induced pro-survival NF-κB signaling, a NF-κB-reporter murine leukemia cell line^36,37^ was incubated in pre-coated wells. Among the adhesion molecules tested, E-selectin was unique in its potential to activate NF-κB reporter activity in reporter cells at a level equivalent to 27 ng mL^−1^ recombinant TNF-α (Fig. 6a). Small synthetic PI3K/AKT antagonist LY29004 (Fig. 6b) or downstream IκB/NF-κB antagonist BMS-345541 (Fig. 6c) were then utilized to confirm the importance of these intracellular pathways to E-selectin-adhesion-mediated chemo-resistance. We found that addition of either NF-κB or AKT inhibitor could reverse the E-selectin-mediated chemoresistance of blasts in vitro (Fig. 6b, c). Together these data suggest that PI3K/AKT/NF-κB pathway is an important mediator of niche E-selectin-induced pro-survival signaling.

To further confirm the relationship between surface receptor fucosylation (E-selectin binding potential) and intracellular signaling, AML blasts generated by MLL-AF9 transduction of HSCs from *Fut4*^−/−^ *Fut7*^−/−^ double gene-deleted mice or matching wild-type control mice, were adhered to precoated wells for 25 min. Immunoblotting using the quantitative linear LI-COR system revealed E-selectin-mediated adhesion induced a threefold increase in Ser473 phosphorylation of AKT in WT AML blasts, but not in AML blasts deleted for *Fut4* and *Fut7* (Fig. 6d). Together these data demonstrate a critical link between AML cell surface *α*(1,3) fucosylation and E-selectin-mediated pro-survival signaling.

Finally, to confirm whether absence or therapeutic blockade of E-selectin dampens pro-survival PI3K/AKT/NF-κB and/or mTOR signaling in AML blasts in vivo, BM from mice with advanced AML (>80% AML blasts) were administered either E-selectin antagonist GMI-1271, or saline vehicle control (experimental outline Fig. 6e). Immunoblotting of BM lysates revealed that both AKT^Ser473^ and NF-κB p65^Ser536^ phosphorylations were halved 24 h after GMI-1271 administration in vivo (Fig. 6f, g). A similar reduction was also observed with wildtype AML blasts in *Sele*^−/−^ hosts (Fig. 6g; Supplementary Fig. 6). As mTOR signaling is also downstream of AKT, phosphorylation levels of p-S6K p70 (Ser371) and p-4E-BP1 (Thr37/46), both part of the mTOR signaling pathway, were also investigated. While a 40–50% reduction in the phosphorylation of p-S6K p70 in AML was observed with absence or blockade of E-selectin, no changes were observed for 4E-BP1 (Fig. 6f, g). Together, these data confirm that absence or blockade of E-selectin dampens pro-survival AKT and NF-kB signaling in BM AML in vivo.

## Discussion

Niche hijack refers to how malignant cells alter their microenvironment to benefit their own expansion and survival at the detriment of host nonmalignant cells^38,39^. In this paper we show endothelial E-selectin to be a key target of niche hijack in AML. We find pro-inflammatory mediators released by AML blasts increases the expression of E-selectin on the BM endothelium thus generating the formation of a protective endothelial niche for AML cell survival.

E-selectin expression has previously been reported at the leading edge of solid tumors^40^ as well as at pre-metastatic sites^41^. This suggests the release of inflammatory mediators by malignant cells and induction of potentially protective E-selectin-expressing vascular niches may be wide-spread. Our data suggest the upregulation of E-selectin on tumor associated vasculature (in contrast to P-selectin) may be less about metastatic cell homing and more as a factor directly contributing to the survival and regeneration of malignant cells such as by the creation of a protective niche and induction of pro-survival (AKT/NF-kB/mTOR pathway) signaling thereby promoting therapy resistance. It has been recently reported that adhesion to E-selectin may induce mesenchymal-to-epithelial transition in breast cancer cells^42^ both these mechanisms may contribute to BM metastasis for solid tumors.

Although we show absence or therapeutic blockade of E-selectin increased AML blast chemo-sensitivity, E-selectin blockade was only associated with a small (<1%) relocation of AML blasts from the BM to the blood. This lack of effect on AML blast re-localization is likely due to redundancy of E-selectin with the range of other adhesion molecules and chemokines involved in BM retention. Indeed P-selectin, as well as integrin ligands such as VCAM-1/CD106 are far more efficient mediators of leukocyte homing^43–45^. Surprisingly in our studies, neither alternative adhesion molecule appeared to mediate AML chemo-resistance, nor initiate intracellular AKT phosphorylation upon AML adhesion. Thus E-selectin appears to be unique among these adhesion molecules in playing a key role in mediating pro-survival signaling in malignant cells.

Under normal conditions only leukocytes have the potential to bind selectins. The generation of complex carbohydrates such as sLe^x/a^ requires the coordinated action of a series of glycosyl-transferases (including beta-galactoside alpha-2,3-sialyltransferase 4 [ST3GAL4] to generate α2-3 sialic acid linkages on position 3 of a galactose-containing glycoprotein or glycolipid) and the α(1,3)-fucosyl transferases to form the sLe^x/a^ lectin binding domain^17,18,46^. However, oncogenesis is often associated with alterations in cellular glycosylation^10,46,47^ leading to de novo, or an increased expression of functional selectin receptors on malignant cells, that are not normally observed on non-malignant counterparts. Herein, we show that acquisition of selectin-binding potential by AML blasts directly enables significant E-selectin-mediated intracellular survival signaling distinct from roles in AML cell homing or metastasis.

The changes in cell surface glycosylation observed with oncogenic transformation are likely mediated by epigenetic mechanisms. Inflammatory mediators (such as TNF-α^48^ and G-CSF^49^) have previously been reported to lead to increased ST3GAL4, FUT4, and FUT7 expression, which are largely regulated by repressive promoter methylation^50,51^. Thus, it is tempting to speculate that the altered glycosylation (and increase in E-selectin binding potential) on malignant cells is associated with the epigenetic changes in DNA methylation and histone modulation that occurs during oncogenesis, possibly re-enforced by tumor-associated inflammation. Interestingly, drug efflux transporter genes such as ABCG2 (BCRP) and MDR1 (P-gp) have also been reported to be regulated by promoter methylation together with histone modifications^52,53^. Thus, the correlation between E-selectin binding and drug efflux potential reported herein may reflect the underlying changes in the methylation status of a malignant cell.

Finally, our data suggest several striking differences between malignant AML blasts and the endogenous nonmalignant HSPC population from which they originated in response to E-selectin binding. These differences include: (i) E-selectin adhesion mediates AML chemo-resistance but not HSPC chemoresistance in vitro, (ii) absence or therapeutic blockade of E-selectin induces AML blasts to leave quiescence and engage in cell cycle whereas in contrast E-selectin blockade dampens HSPC cycling, (iii) AML blasts highest for E-selectin binding potential contain all drug efflux capacity whereas HSPC efflux equally well regardless of E-selectin binding potential, and (iv) E-selectin adhesion mediates pro-survival AKT/NF-κβ/mTOR signaling in AML blasts but not in HSPC nor in AML blasts unable to bind E-selectin due to fucosyl transferase FUT4/7 deletions.

In conclusion, we describe a form of niche hijack whereby release of inflammatory mediators induced by malignant cells leads to an upregulation of adhesion molecule expression on niche endothelium and E-selectin expression. Contact with E-selectin directly mediates malignant cell regeneration, survival and chemo-resistance via triggering of pro-survival intracellular signaling such as AKT/NF-κB/mTOR pathways well-known to mediate therapy resistance. We report therapeutic E-selectin blockade in vivo dampens this pro-survival signaling, effectively neutralizing the deleterious effects of this niche hijack leading to significantly improved therapeutic outcomes in mice. As de novo or increased E-selectin binding potential is reported in a wide variety of malignant cells^21,54^, as is the upregulation of endothelial E-selectin expression^40,41^, this form of niche hijack and E-selectin mediated therapy-resistance may be wide-spread among diverse tumors.

The data in this paper are experimental basis for a phase I/II clinical trial (NCT02306291) evaluating the use of GMI-1271/Uproselesan, adjunct to standard therapy in relapsed/refractory (r/r) adult AML. This trial reported administration of adjunct GMI-1271 therapy associated with higher rates of clinical remission and extended median patient survival compared to comparable historical cohorts and consistent with our preclinical mouse studies reported herein. Two Phase II/III clinical trials further evaluating the efficacy of GMI-1271/Uproselesan as adjunct therapy in newly diagnosed (NCT03701308) and relapsed/refractory (NCT03616470) adult AML are currently underway.

## Methods

### Mice, ethics, and tissue processing

All mouse experiments were approved by The University of Queensland animal ethics committee. C57BL/6 mice (CD45.2^+^) and congenic B6.SJL (CD45.1^+^) were purchased from Animal Resource Centre, Perth, Australia. Mice were housed in Tecniplast Greenline IVC caging in a room with controlled temperature (22–26 °C) and humidity (30–70%). All experimental groups were age- and sex-matched (10–16 weeks). Generally, donors were males and recipients females. *Sele*^−/−^ mice were a kind gift from P. Frenette (Albert Einstein College of Medicine, NY) and *Fut4*^−/−^ *Fut7*^−/−^ double KO mice from J. Rasko. All strains had been backcrossed on C57BL/6 background at least ten generations.

Heparinized blood was collected from anaesthetized mice by cardiac puncture then spleens, leg bones, and occasionally spines collected. Femurs were generally flushed in 1 mL ice-cold phosphate buffered saline (PBS) plus 2% newborn calf serum (NCS) using needle and syringe for the isolation of whole BM cells (~25 × 10^6^ cells per femur). For some experiments BM AML blasts were further enriched from BM using mouse CD117-conjugated Magnetic Activated Cell Sorting (MACs) beads and “POSSEL” positive selection on auto-MACS (Miltenyi, Germany) as previously described^6^.

For endothelial cell stains, endosteal cells were harvested from already flushed femurs as outlined above. After additional washing with PBS, these bones were gently flushed using a syringe and 23 G needle with 0.2 ml pre-warmed collagenase type 1 (Worthington, #CLS-1) diluted to 1 mg mL^−1^ in IMDM, for 20 min with agitation at 37 °C to release endosteal cells which were then collected by flush with PBS and analyzed by flow cytometry^6^.

Collection and staining of healthy patient BM HSC and leukemia cells were approved by the Peter MacCallum Cancer Centre ethics committee (Project 01/40) and Queensland Metro South Human Ethics Committee (HREC/2019/QMS/46799). All collections were from consenting adult patients that sequentially presented to clinic. Donor BM cells were collected from normal healthy BM donors (*n* = 2) or from BM associated with acetabulum reaming from patients undergoing arthroplasty (*n* = 2). AML cells were collected from blood or BM of patients at the same time as standard of care collection (*n* = 2). Mononuclear cells were enriched for CD34^+^ cells using anti-CD34 conjugated MACs beads and auto-MACs POSSEL separation following manufacturer’s instructions. Cells were then stained for flow cytometry.

Calculation of whole body AML blast numbers in BM and blood for Supplementary Fig. 3 were performed as previously described^55^. In summary one femur represents 5.6% total BM, and total blood volume of a mouse calculated as 0.8 mL peripheral blood per 10 g body weight.

### Reagents

Dulbecco-modified Eagle’s medium (DMEM), Iscove-modified Dulbecco’s medium (IMDM), minimum essential medium (MEM)-α (without phenol red), Dulbecco’s PBS (DPBS), and fetal calf serum (FCS) were purchased from ThermoFisher. X-VIVO-10 and X-VIVO-15 media were from Lonza. Polybrene and ethylenediaminetetraacetic acid (EDTA) were purchased from Sigma-Aldrich. 7-aminoactinomycin D (7AAD) was purchased from Molecular probes, Life technologies. PI3K inhibitor LY294002 and selective inhibitor of IκB kinase/NF-κB signaling BMS-345541 were from Selleckchem. Neutralizing rat anti-mouse E-selectin monoclonal antibody (clone RME-1) from BioLegend was used at 10 μg mL^−1^ for blocking. Recombinant mouse adhesion molecules E-selectin-huIgG1 Fc, P-selectin-huIgG1 Fc, CD31/PECAM1-huIgG1 Fc, VCAM1-huIgG1 Fc, ICAM1-huIgG1 Fc, and negative control CD14-huIgG1 Fc were all from R&D Systems. Recombinant mouse stem cell factor (SCF), human interleukin 6 (IL-6), and mouse IL-3 were from Peprotec. Cytarabine and doxorubicin were sourced from the Mater Hospital Pharmacy. Clinical grade GMI-1271 (Uproselesan) was provided by GlycoMimetics, Rockville, MA.

### Initiation of AML in mice

Bicistronic MSCV retroviral vector carrying GFP and the human MLL-AF9, MLL-ENL, or AML1-ETO9a fusion oncogenes were kindly provided by Scott Lowe^8^. pMSCV-MLL-AF9-ires-GFP and empty vector control were transfected into GP + E86 ecotropic retrovirus packaging cells and 3 days later, GFP^+^ packaging cells were purified by fluorescence-activated cell sorting (FACS) and expanded. For murine HSPC transfection, packaging cells were first irradiated (15 Gy), then cocultured together with FACS sorted Lineage (CD3ε, CD5, Ter119, B220, and Gr-1)^neg^ CD11b^−^ KIT^+^ Sca-1^+^ (LSK) cells collected from mouse BM 7 days post 5-fluorouracil induction (150 mg kg^−1^ intraperitoneal injection). Medium for transduction (DMEM) containing 10% FCS, 5 μg mL^−1^ polybrene plus cytokines, 50 ng mL^−1^ recombinant mouse (rmu) SCF, 10 ng mL^−1^ recombinant human (rhu) IL-ll, and 10 ng mL^−1^ rmu thrombopoietin (Peprotech). After 24–72 h, transduced HSPC were retrieved from the coculture by gentle washing, transferred to new cell culture flask for 2–4 h to ensure no adherent packaging cells remained, then washed in saline before retro-orbital intra-venous injection into conditioned (4 Gy) primary recipient mice for AML to develop. As a control for transduction efficiency, a portion of the transduced LSK cells were also cultured an additional 3 days and analyzed by flow cytometry for GFP expression. Typical transduction efficiency was 10%.

Emergence of AML in recipient mice was monitored by flow cytometry from regular tail vein test-bleeds for appearance of GFP^+^ KIT^+^ AML blasts. Once over 40% of peripheral blood CD45^+^ leukocytes were GFP^+^ KIT^+^ (typically 30–60 days post transplantation) mice were euthanized and ~10^4^ BM cell aliquots (typically >80% AML blasts) used for transplantation via the retro-orbital sinus into large cohorts of matched 2.5 Gy conditioned recipient mice for experimental procedures.

### Co-culture of BMEC-1 with murine whole BM cells

Human Bone Marrow Endothelial Cell line BMEC-1 (ATCC #CRL-3421) were seeded at 10,000 cells per well of a 96-well tissue culture plate in 100 μL of αMEM supplemented with 10% FCS, 1× penicillin streptomycin glutamine (PSG, Gibco) and 10 ng mL^−1^ rmu epidermal growth factor (rmuEGF, Biolegend, cat#585606). After 7 h of incubation (37 °C, 5% CO_2_), fresh mouse whole BM cells were added to the wells and medium was replaced with fresh αMEM supplemented with 10% FCS, 1× PSG, 10 ng mL^−1^ rmuSCF and 10 ng mL^−1^ rmuEGF. In some wells, rmuTNF-α (Biolegend) was added at a final concentration of 10 or 30 ng mL^−1^ instead of whole BM cells as a positive control for E-selectin expression. In some wells, etanercept (Enbrel®, AMGEN) was added at a final concentration of 1 μg mL^−1^ as indicated. BM cells were cocultured with BMEC for 16 h (37 °C, 5% CO_2_), then collected by gentle pipetting on ice to retrieve cells and stained with APC-conjugated mouse anti-human E-selectin (Biolegend, cat# 336012). Cells were then analyzed by flow cytometry in the presence of 1.7 μg mL^−1^ 7AAD for live/dead exclusion. Gating strategy and dot plots from viable cells are shown in Fig. 1e.

### In vivo assays

GMI-1271 was administered to mice intraperitoneally at 40 mg kg^−1^ per injection bidaily (BiD) for 5 days or at higher doses for shorter 24 h readouts as indicated. For AML cell mobilization, mice were administered a single dose of 40 mg kg^−1^ GMI-1271 retro-orbitally followed by regular test bleeds (2 drops of blood from lateral tail vein into heparin) at indicated time points before and after injection. Twelve microlitre of whole blood was taken for leukocyte count (Hematology Analyzer Coulter Ac•T diff, Beckman Coulter) and the remainder subject to red cell lysis (NH_4_Cl) before staining for flow as described below.

Cytarabine was administered as single agent at high-dose (two injections each 900 mg kg^−1^ intraperitoneally 12 h apart, with euthanasia 24 h after first injection). Alternatively for disease-free survival studies, cytarabine was administered in combination with doxorubicin for a total of 5 days (cytarabine 100 mg kg^−1^ intravenous daily for 5 days and doxorubicin 1 mg kg^−1^ daily intravenous injections for first 3 days).

### Flow cytometry

Supplementary Table 1 lists antibodies used for flow cytometry and immunoblotting studies. Endothelial cells from the BM endosteal region were identified by flow cytometry as negative for lineage markers (CD3ε, CD5, Ter119, B220, and Gr-1)-PerCPCy5.5, CD11b^−^PerCPCy5.5, GFP^−^, CD45^dim to neg^ APCCy7, and CD31^+^ APC together with phycoerythrin (PE)-conjugated rat anti-mouse E-selectin (clone UZ6)^6^. Analysis of E-selectin binding potential was performed using recombinant human-E-selectin-human–IgM fusion protein (plasmid kind gift from Karen Snapp, University of Illinois, IL) pre-complexed with affinity purified donkey F(ab)_2_ anti-human IgM conjugated to CY5, AF647, or BV785 (Jackson ImmunoResearch, PA)^6,56^. This complex was incubated with the cells in 10 μL volume for 20 min on ice, then washed and resuspended in XVIVO-10 for flow cytometry analysis.

Human BM HSCs were identified as negative for lineage (CD3, CD19)-PacificBlue, CD33^−^BV421, CD34^+^ PE, CD38^−^PECy7 cells whereas murine HSPCs were defined as Lineage^−^ (CD3ε, B220, Ter119, Gr-1)-PerCP-Cy5.5, CD11b^−^PacificBlue, KIT^+^APCCy7, Human AML blasts were identified as CD33^+^ BV421 CD34^+^ PE and murine AML blasts as GFP^+^ KIT^+^ APCCy7 CD11b^+^PacificBlue, Lineage^−^ (CD3ε, B220, Ter119, Gr-1)-PerCP-Cy5.5.

For Rhodamine-based dye efflux studies, 10^6^ murine BM cells were resuspended in 400 μL of X-VIVO 15 serum-free medium containing 10 nM TMRM (Invitrogen), and incubated for 20 min at 37 °C. Cells were washed twice in X-VIVO 15 medium, resuspended in fresh warm 400 μL X-VIVO 15 medium and incubated at 37 °C for a further 15 min to allow dye cellular efflux. After efflux, cells were pelleted, and stained on ice with antibodies to Kit-APCCy7, Sca1-BV510, Lineage (CD3ε, CD5, Ter119, B220, and Gr-1)-PerCP-Cy5.5, CD11b-PacificBlue, and FVS700 (Fixable Viability Stain) before addition of conjugated E-selectin-IgM-biotin-streptavidin-BV785 (to assess for E-selectin binding potential). For all samples a parallel stain exactly as described above was performed, but with addition of 25 µg mL^−1^ verapamil (Isoptin®, Abbott Australasia) throughout for “no efflux” control.

Data were collected using BD FACS ARIA III Fusion, CytoFLEX S Beckman Coulter and CYAN ADP Beckman Coulter. All data were imported as uncompensated files and re-compensated. Data were analyzed using softwares: Summit, FlowJo 7 (Cyan), CytExpert 2.2.0.97, FlowJo 10 (CytoFlex), and Diva 6, FlowJo 10 (Aria).

### BrdU incorporation and cell cycle analysis

*Sele*^−/−^ and age-matched, sex-matched C57BL/6 mice (CD45.2^+^) mice were transplanted with 10^4^ BM KIT^+^ leukemic blasts generated from transduced B6.SJL (CD45.1^+^) murine HSC. The use of a CD45.1^+^ AML was required in this experiment as GFP fluorescence (our usual marker for transduced AML) is lost after fixation. Cohorts of mice were administered GMI-1271 (40 mg kg^−1^ BiD, intraperitoneally) or saline vehicle from day 16 to 20 post-transplant, and all mice were administered 0.5 mg BrdU intraperitoneally at day 20 post-transplant, exactly 24 h prior to euthanasia.

Two million whole-BM cells were stained with anti-CD45.1-FITC, Lineage (B220, CD3, CD5, Gr-1, and Ter119)-PerCP-Cy5.5, anti-Sca1-PE-CY7, anti-KIT-APC, and anti-CD11b-PacificBlue. Cells were then fixed and permeabilized using the BD Pharmingen BrdU kit (cat# 51-9000019AK) before staining with antibodies to anti-BrdU-PE and anti-Ki67-AF700 monoclonal antibodies^6^. Representative dotplots and gating strategy are shown in Fig. 4a.

### In vitro chemosensitivity and reporter assays

Wells of 96-well tissue culture plates (Nunc^TM^, ThermoFisher) were coated with 5 µg mL^−1^ recombinant adhesion molecules diluted in Tris-buffered saline (TBS) pH 8.0 overnight at 4 °C, then wells washed and blocked with 0.5% bovine serum albumin in PBS for 1 h at 37 °C as described previously^56^. KIT^+^ MACS-enriched BM AML blasts from MLL-AF9 or MLL-ENL transduced leukemic mice, or matching KIT^+^-enriched healthy BM cells (from non-leukemic mice), were cultured for 5–10 days in IMDM complete medium plus 10% FCS in the presence of cytokines (20 ng mL^−1^ rmuSCF, 10 ng mL^−1^ rhuIL-6, and 10 ng mL^−1^ rmuIL-3) then seeded into pre-coated 96-well plates (at 10,000 AML blasts per 100 µL well) in XIVO-10 medium (Lonza Bioscience) with cytokines as above. After 6 h incubation for cell adhesion, cytarabine (final 25 ng mL^−1^ as selected dose by limiting dilution), or saline vehicle control was added to wells then plates incubated a further 66 h at 37 °C. To count cells at endpoint, wells were washed gently with TBS and cells resuspended by vigorous pipetting in presence of cold EDTA (10 mM final). The number of viable GFP^+^ AML cells per well was then counted by volumetric flow cytometry on CytoFlex S flow cytometer (Beckman Coulter) in the presence of 1.7 μg mL^−1^ 7AAD for viability gating. In some studies, LY294002 (final 2.5 μM) and BMS-345541 (final 10 μM) were added after initial 6 h incubation with adhesion molecules. A similar protocol was used with human CD34^+^ AML cell line KG1a but with an earlier readout (48 h) after addition of 10 μg ml^−1^ cytarabine.

The mouse monocytic cell line RAW264.7 (from ATCC) derived from a Murine Leukemia Virus injected mouse^37^ stably transfected with NF-κB response element from the *SELE* gene promotor driving GFP reporter expression^36^ was used to study NF-κB activation in live cells in response to cell adhesion. NF-κB reporter RAW264.7 cells were added to pre-coated wells of non-tissue culture treated 96-well plates (Iwaki, Japan) at 100,000 cells per 100 µL well on ice in the presence of 10 µM BMS-345541 or recombinant mouse TNF-α (Biolegend) dilutions. Following a brief centrifugation (200*g*, 1 min) to bring cells into contact with pre-coated surfaces, cells were incubated at 37 °C for 3 h in contact with pre-coated surface. Reporter cells were then resuspended by gentle pipetting and analyzed by flow cytometry for GFP fluorescence indicative of NF-κB mediated transcription in the viable 7AAD^−^ fraction.

### In vivo leukemia stem cell survival assay

Mice with advancing AML (5–20% GFP^+^ KIT^+^ AML blasts in blood, typically ~3.5–4 weeks post-transplant) were administered two high dose rounds of cytarabine (900 mg kg^−1^ intraperitoneally) at 24 and 16 h prior to euthanasia. Femurs were collected and crushed in PBS with 2% NCS. Serial dilutions of BM cells (final 1%, 0.1%, 0.01% of one femur) were transplanted into 2.5 Gy conditioned C57BL/6 recipient mice. At 4 weeks post-transplant recipient mice with over 5% GFP^+^ KIT^+^ AML blasts in blood (corresponding to >40% GFP^+^ KIT^+^ in BM in post-mortem analyses) were considered AML ‘positive’. Poisson distribution statistics (L-calc software, Stem Cell Technologies, Canada) were then used to calculate frequency of LRC among femoral BM cells ±95% confidence interval. For figure generation, total LRC numbers per femur were calculated by multiplying frequency of LRC with total BM cell number. Comparative statistically analysis of calculated LRC frequency ±95% CI between the different experiment cohorts was also determined using Lcalc software.

### Disease-free survival study

Cohorts of matched C57BL/6 (10-week-old males) mice transplanted with 10^4^ BM KIT^+^ leukemic blasts were administered GMI-1271 (40 mg kg^−1^ BiD intraperitoneally) from day 6 to day 15 post-transplant. At day 10 post-transplant, mice received induction chemotherapy (5 days cytarabine at 100 mg kg^−1^ intraperitoneally plus doxorubicin 1 mg kg^−1^ intravenously for the first 3 days). Treated mice were monitored daily for recovery, wellbeing and time to relapse (defined as >50% GFP^+^ KIT^+^ leukemic blasts in blood; >80% in the BM).

### Immunoblotting

In vitro adhesion: BM MLL-AF9 KIT^+^ blasts from advanced leukemia mice were washed in PBS, resuspended in X-VIVO medium and placed in 96-well tissue culture plate wells, pre-coated with recombinant cell adhesion molecules, on ice (200,000 cells per 100 µL well) followed by a quick one minute 200*g* centrifugation at 4 °C to bring cells into contact with pre-coated surface. Plates were then rapidly brought to 37 °C by placing on a pre-warmed heating block before transfer to a 37 °C incubator. After 25 min at 37 °C, plates were placed on ice to stop signaling, supernatant removed and adherent cells lysed in 100 µL of TBS with 1% NP-40 as lysis buffer supplemented with protease (#04693159001) and phosphatase (#04900837001) inhibitors PhosStop from Roche, Mannheim, Germany. After 10 min lysis on ice, cell lysates were transferred to microfuge tubes and centrifuged 12,000*g* for 5 min at 4 °C. Nuclei free supernatants were then mixed (4:1 ratio) with sample loading buffer containing 10 mM final dithiothreitol and heated (95 °C, 1 min). Twenty microlitre cell lysate (containing proteins derived from ~30,000 AML blasts) and pre-stained protein markers (Novex, Invitrogen) were loaded to each lane and proteins separated on a 4–12% gradient SDS-PAGE gel (#NW04125BOX) before transfer onto nitrocellulose membrane (IBC3002 Invitrogen, ThermoFisher Scientific, MA, USA). Quantification of total transferred protein per lane was performed by REVERT stain (TBS cat# 927-50000, Li-Cor, NE, USA) following manufacturer’s instructions. Membranes were blocked with Odyssey blocking buffer (Li-COR) and subsequently probed with various monoclonal rabbit antibodies (Supplementary Table 1) and murine pan anti-β-actin antibody (#NB600-501, Novus) for loading control. This was followed by secondary staining with IRD800-conjugated donkey anti-rabbit IgG (H + L) (#5151) together with DyLight680 conjugated goat anti-mouse IgG (H + L) (#5470 P) (both from Cell Signaling Technologies) for parallel detection of house-keeping mouse β-actin. Antibody binding was quantified on the Odyssey Infrared Imaging System (Li-COR) equipped with 2 solid-phase lasers at 700 and 800 nm. Membranes were then stripped using Restore PLUS stripping buffer (#46428, ThermoFisher) for 10 min at room temperature and following a check that no primary antibody remained, the blots were restained with anti-pan AKT antibody. Final Image analysis and band quantifications were performed using Image Studio Lite software (version 5.2). For fold-change calculations, the readout intensity of each specific band was first corrected against matching (β-actin) house-keeping control.

### In vivo signaling

Tibia were removed from leukemic mice, both ends cut with scalpel and BM flushed once into microfuge tubes using 1 mL syringe containing 500 µL urea lysis buffer (7 M urea, 10% glycerol, 1% sodium dodecyl sulfate, 5 mM EDTA, 20 mM Tris-HCl, pH 6.8) with complete protease and phosphatase inhibitor cocktails (Roche) and placed immediately on dry ice as previously described^57^. Following thawing, BM cell lysates were sonicated (3 pulses each 5 s) with probe tip on ice to break up genomic DNA, centrifuged 10,000*g* in 4 °C microfuge for 15 min to pellet debris then debris-free fraction collected. After protein concentration determination by Bradford protein assay (BCA, #23225 Pierce), BM lysates were mixed (4:1) with 5× electrophoresis loading buffer and 10 mM final dithiothreitol and boiled for 3 min. A total of 15 μg whole protein was loaded per lane for immunoblotting and bands separated using a 4–12% gradient SDS-PAGE gel and transferred to PVDF membrane. Transferred proteins were quantified using REVERT stain. Blocking, staining and analysis were same as described above using antibodies listed in Supplementary Table 1.

All unprocessed gel images are provided as a Source Data file.

### Statistics

GraphPad Prism software version 7 was used for statistical analyses. Unless otherwise indicated, groups were compared using One-way ANOVA, multiple comparisons with Bonferroni correction. Survival curves were analyzed using log-rank (Mantel–Cox) test. Limiting dilution analysis were performed using Poisson distribution statistics with L-calc software (Stem Cell Technologies). Two-tailed paired *t* test was used in Figs. 2b and 5e as indicated in Figure legend. All bars show mean with standard deviation unless otherwise indicated.

### Reporting summary

Further information on research design is available in the Nature Research Reporting Summary linked to this article.

## Supplementary information

Supplementary Information

Reporting summary

## Data Availability

Full unprocessed scans of the gels and blots from Fig. 6 and Supplementary Fig. 6 are available in the Source data file. The source data underlying Figs. 1a, b, d–f, 2a–c, 3a–f, 4a, b, 5a, 5c–e, 6a–f, Supplementary Figs. 2, 3, 4, 5a, b, and 6, are provided as a Source data file. All the other data supporting the findings of this study are available within the article and its supplementary information files and from the corresponding author upon reasonable request. A reporting summary for this article is available as a Supplementary Information file.

## Source Data

Source data file
