# Supplementary material for: Endothelial E-selectin inhibition improves acute myeloid leukaemia therapy by disrupting vascular niche-mediated chemoresistance
Source: Nat Commun. 2020 Apr 27;11:2042. doi: 10.1038/s41467-020-15817-5 (PMC7184728; doi:10.1038/s41467-020-15817-5)
Supplement: Supplementary file 1 — Supplementary Information [file 41467_2020_15817_MOESM1_ESM.pdf]

Supplementary Information

Vascular niche hijack via E-selectin promotes niche-mediated acute myeloid leukemia stem cell chemoresistance and regeneration

Barbier and Erbani et al.

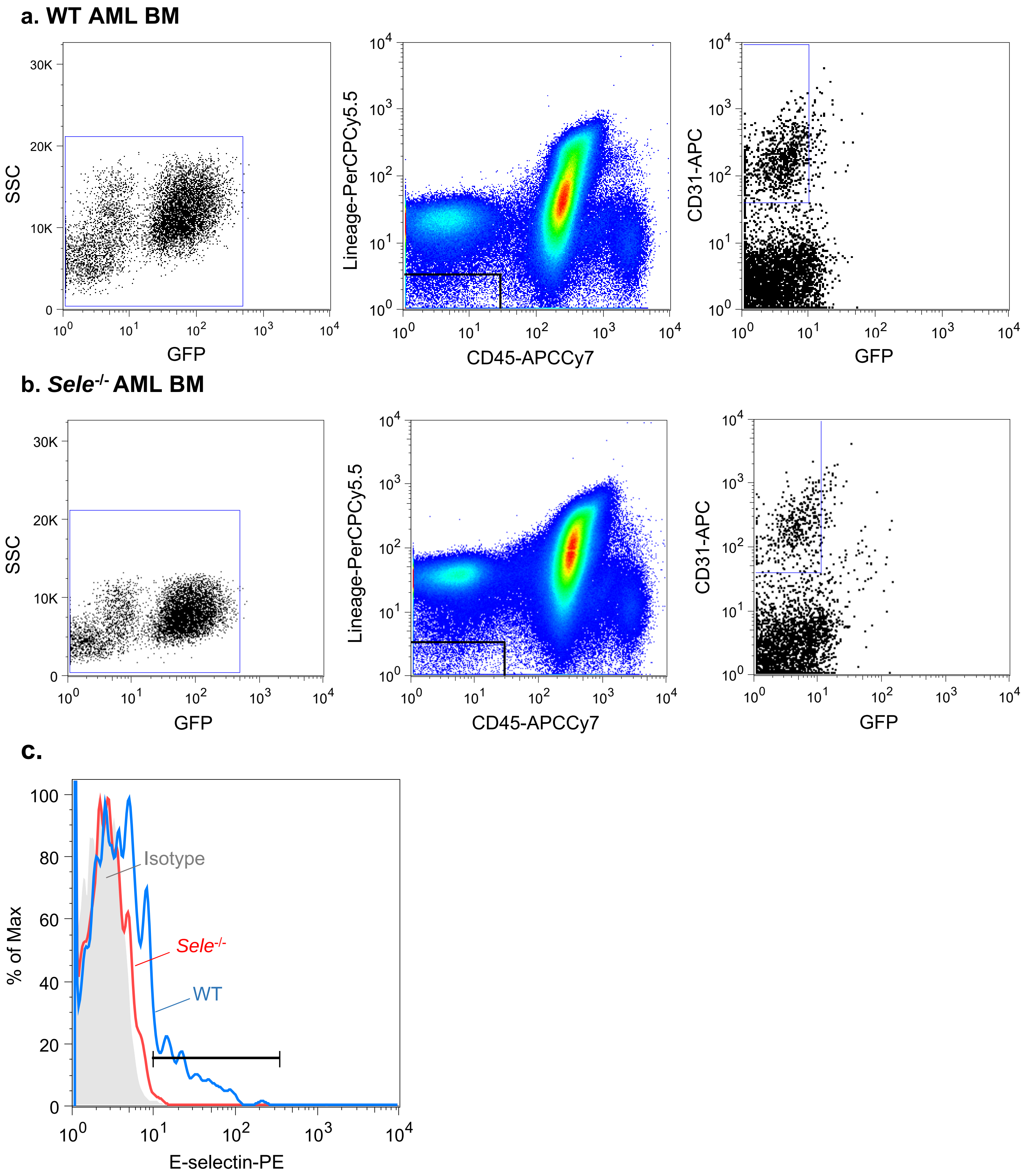

Supplementary figure 1. Endothelial cells from E-selectin KO (*Sele*$^{-/-}$) leukemic mice do not express E-selectin. Endosteal BM was collected from WT or *Sele*$^{-/-}$ mice with advanced GFP^{+} AML (MLL-AF9 induced, n=5 / group) as described in methods, stained for E-selectin expression on surface of viable endothelial cells (7AAD- lineage- CD45low CD31+ GFP-) and analyzed by flow cytometry as previously described. (a-b) Bone marrow endothelial cell gating strategy from one representative mouse in each group: (a). WT leukemic mouse, (b) *Sele*$^{-/-}$ leukemic mouse. (c) Overlay histogram comparing cell surface E-selectin expression on gated BM endothelial cells from WT (blue line) or *Sele*$^{-/-}$ (red line) leukemic mice. Isotype control is grey filled. Bar shows E-selectin positive gate.

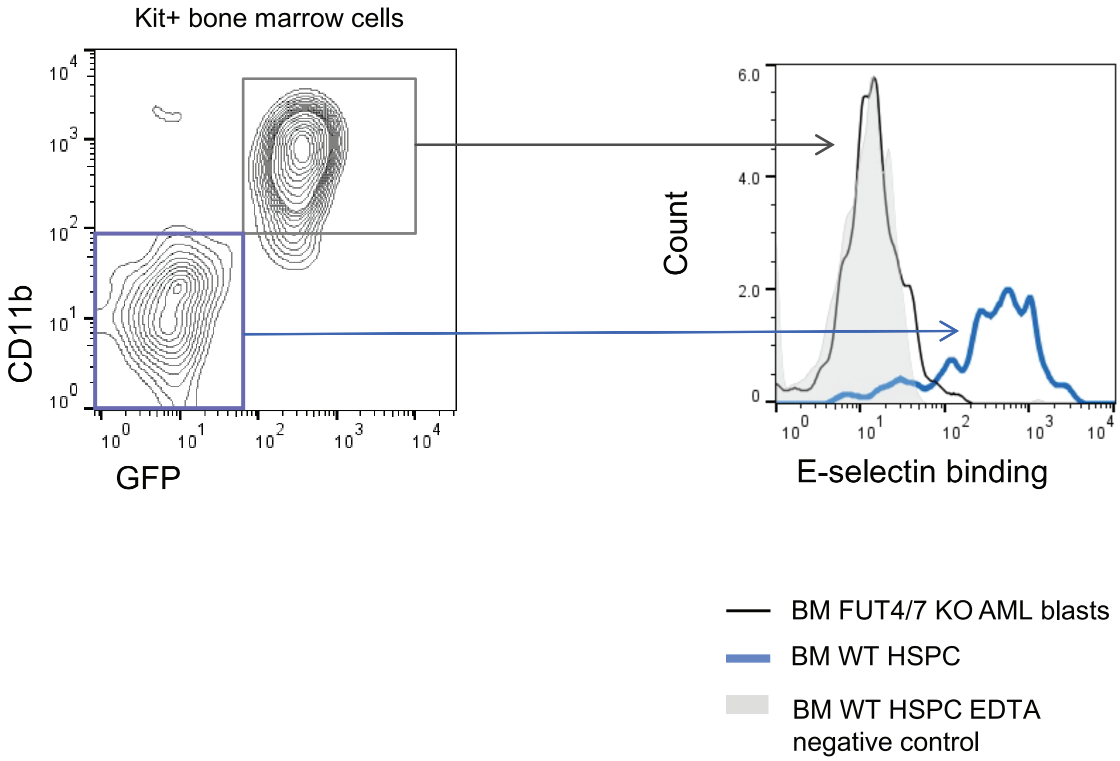

Supplementary Figure 2. AML cells gene-deleted for fucosyltransferase Fut4 and Fut7 no longer bind E-selectin. HSPCs from wildtype or Fut4$^{-/-}$ Fut7$^{-/-}$ (FUT4/7) double gene-deleted mice were transduced with MSCV-MLL-AF9-ires-GFP retrovirus and transplanted into recipient mice for leukemia development. Bone marrow cells collected from wildtype hosts carrying FUT4/7 double gene-deleted AML were analyzed by flow cytometry for E-selectin binding potential. Left panel shows gating strategy within the lineage (B220, CD3ε, Gr-1, Ter119)-negative gate. Shown are c-KIT+ normal wildtype host HSPC (Kit+ GFPneg CD11bneg) and Fut4-/- Fut7-/- AML blasts (Kit+ GFP+ CD11b+). Right panel shows relative E-selectin binding potential of the gated normal wild-type HSPCs (blue line) and adoptively transferred Fut4$^{-/-}$ Fut7$^{-/-}$ AML blasts (black line). Filled grey is negative (non-binding) control made of wild-type HSPCs incubated with E-selectin-IgM construct in the presence of 10 mM EDTA).

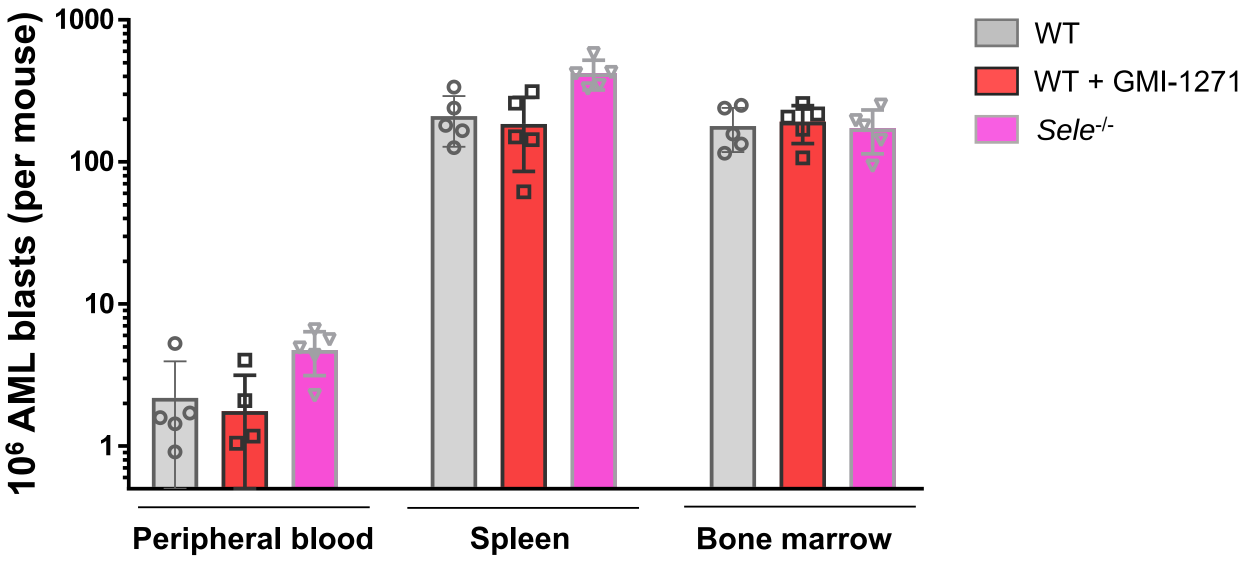

Supplementary Figure 3. Number of AML blasts in peripheral blood compared to other hematopoietic organs. Numbers of AML blasts in peripheral blood, spleen and BM were measured in leukemic wildtype mice administered ± 200mg kg^{-1} GMI-1271 (BiD for 48h) or *Sele*$^{-/-}$ mice. Histogram represents number of AML blasts per mouse in peripheral blood, spleen and bone marrow. Each dot represents data from an individual mouse. All data shown as mean ± S.D (n=5 mice/group). Statistical significance was calculated by one-way ANOVA with Bonferroni correction for multiple comparisons.

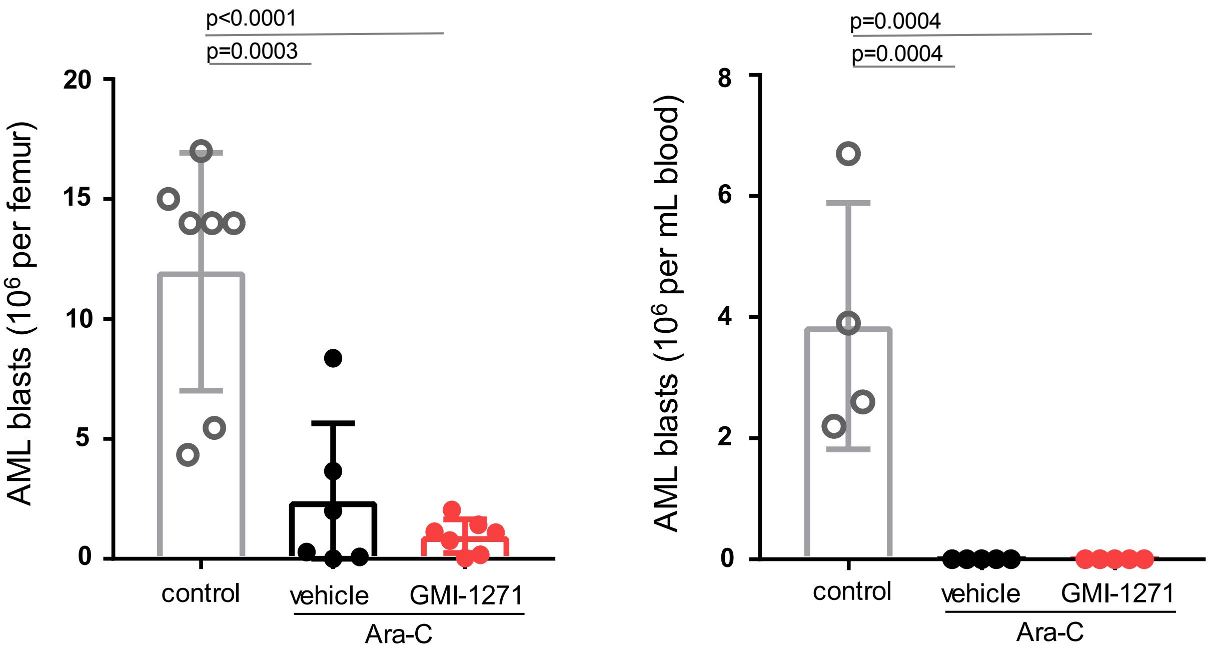

Supplementary Figure 4. Cytarabine treatment ablates all AML blasts in peripheral blood and most (but not all) AML blasts in the bone marrow by 24 hours. Shown are the number of surviving Kit+GFP+ AML blasts per femur (left panel) or per mL blood (right panel) at 24 hours after administration of high dose cytarabine (900mg kg^{-1} BiD) ± GMI-1271 (20mg kg^{-1} BiD for 4d) using experimental plan outlined in manuscript Figure 3c. Each dot represents result from an individual mouse. Bars are mean and SD. n=7 mice / group for bone marrow and n=4,5,5 / group for blood. Statistical analysis; One way ANOVA with multiple comparison Bonferroni correction.

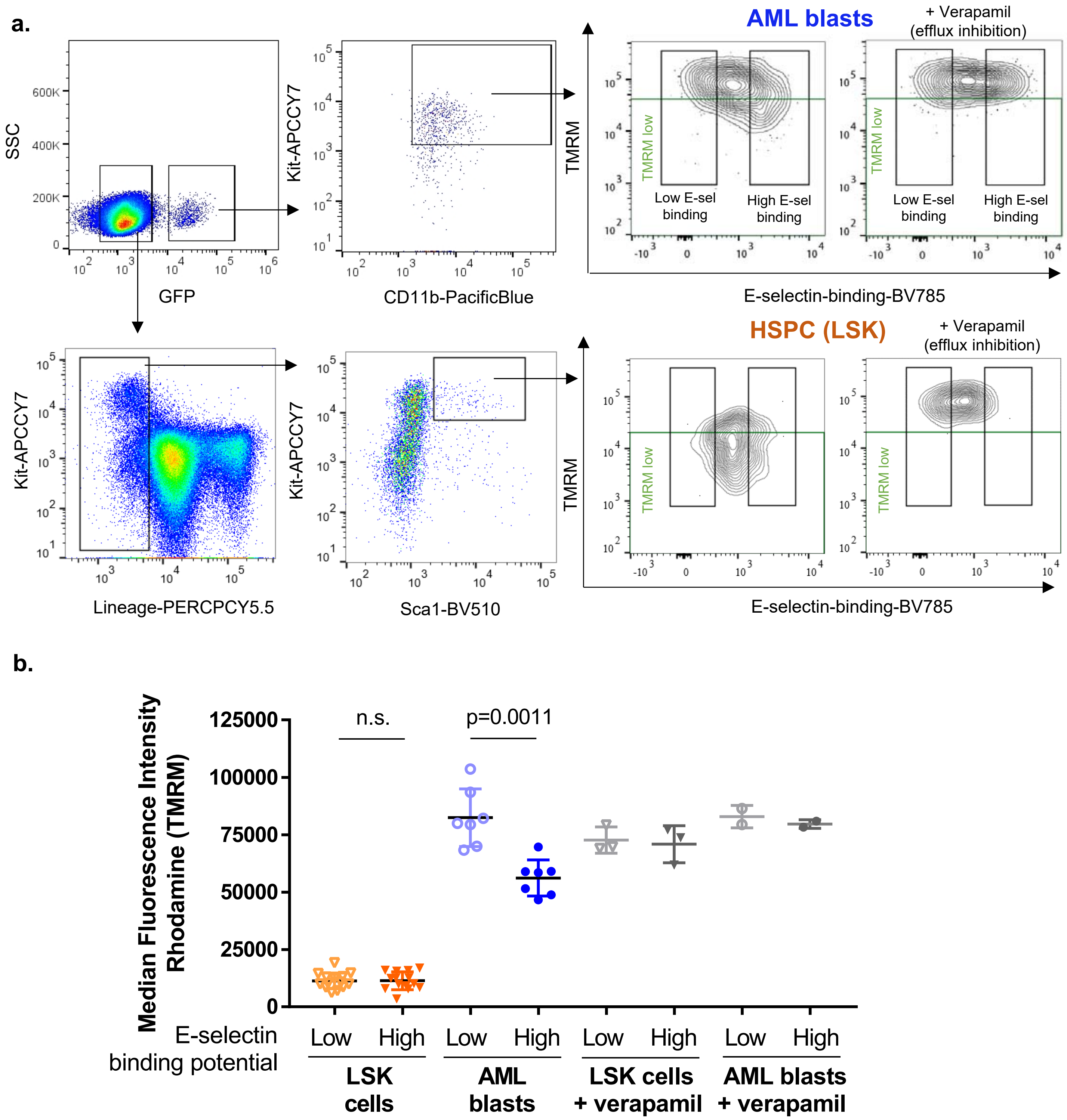

Supplementary Figure 5. E-selectin binding potential correlates with ability to efflux Rhodamine dye in AML blasts, but not HSPCs. (**a**) Dot plots show gating strategy for rhodamine-derivative TMRM efflux by AML blasts (top panel) or HSPCs (bottom panel). Contour plots show TMRM efflux and E-selectin-binding staining on gated bone marrow $GFP^{+}Kit^{+}CD11b^{+}$ AML blasts (top panel) or $GFP^{-}Lin^{-}Kit^{+}Sca\text{-}1^{+}$ HSPC (LSK, bottom panel) from representative mice. Low and high E-selectin binding populations were gated as bottom and top 30%, respectively. AML blasts with the ability to efflux TMRM ("TMRM low" gate) were identified based on samples treated *in vitro* with verapamil (right panel) to block TMRM efflux ("no efflux" control). (**b**) Histogram shows median fluorescence intensity (MFI) of TMRM dye (PE channel) in AML blasts (orange) or LSK cells (blue) ± 50μM verapamil to block TMRM efflux ("no efflux" control). Low MFI values correspond to a high efflux capacity. Data shown as mean ± S.D. from n= 7 (AML) or 14 (LSK) mice per group. Statistical significance was calculated by two-tailed paired t-test.

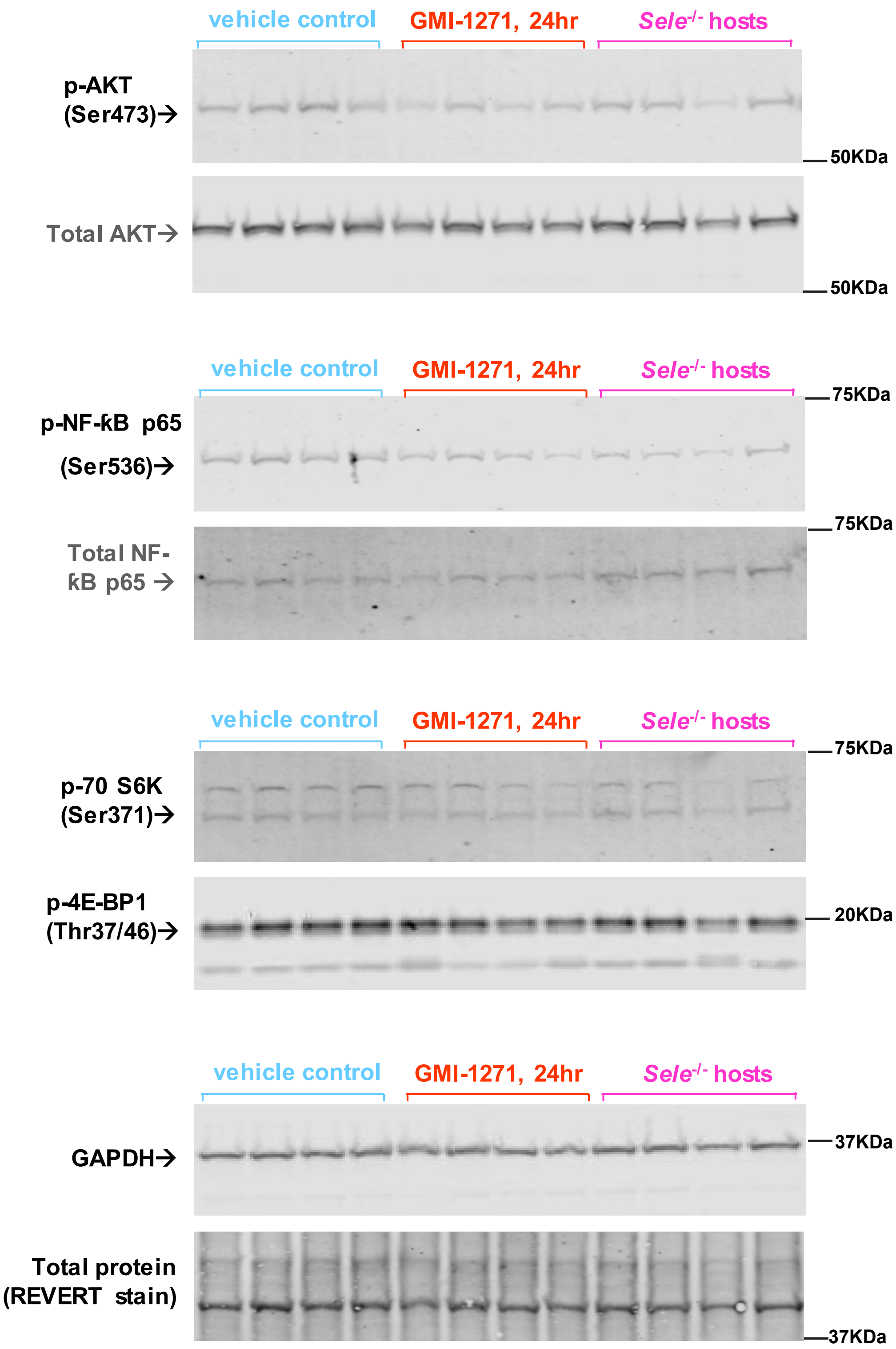

Supplementary Figure 6. Absence or blockade of E-selectin in vivo dampens AKT/NF-kB/mTOR signaling in AML blasts in vivo. Blots from BM cell lysates of wildtype mice administered ± 200mg kg^{-1} GMI-1271 BiD I.P or *Sele*$^{-/-}$ mice each inoculated with wildtype MLL-AF9. These blots were performed as, and are complementary to, those described in manuscript Figure 6.

Supplementary table 1: Antibodies used for flow cytometry and immunoblotting

FLOW CYTOMETRY

Anti-human

**Antibodies**	**Clones**	**Suppliers**	**Dilution**
CD3-PacificBlue	UCHT1	Biolegend	1/50
CD19-PacificBlue	HIB19	Biolegend	1/50
CD33-BV421	WM53	BioLegend	1/40
CD34-PE	561	BioLegend	1/10
CD38-PECY7	HIT2	BioLegend	1/40
IgM-AF647	Polyclonal	Jackson ImmunoResearch	N/A
CD62E-PE	HCD62E	BioLegend	1/20
CD62E-APC	HAE-1f	BioLegend	1/20

Anti-mouse

**Antibodies**	**Clones**	**Suppliers**	**Dilution**
B220-PerCPCy5.5	RA3-6B2	BioLegend	1/250
CD3- PerCPCy5.5	145-2C11	BioLegend	1/250
CD5-PerCPCy5.5	53-7.3	BioLegend	1/250
Ter119- PerCPCy5.5	TER-119	BioLegend	1/250
Gr1- PerCPCy5.5	RB6-8C5	BioLegend	1/300
Sca1-PECY7	D7	BioLegend	1/150
Sca-1-BV510	D7	BioLegend	1/100
CD48-Pacific Blue	HM48-1	BioLegend	1/300
CD150-PE	TC15-12F 12.2	BD Pharmingen	1/200
Kit(CD117)-APCH7	2B8	BD Pharmingen	1/200
Kit(CD117)-PE	2B8	BioLegend	1/200
Kit(CD117)-APCCY7	2B8	BioLegend	1/200
Kit(CD117)-APC	2B8	BioLegend	1/200
CD31-APC	390	BioLegend	1/200
CD48-PerCPCY5.5	HM48-1	BD Pharmingen	1/300
CD45.1-PE	A20	BD Pharmingen	1/200
CD45.1-FITC	A20	Biolegend	1/200
CD45.2-APC	104	Biolegend	1/200
CD45-APCCY7	30F11	Biolegend	1/200
CD11b-PECY7	M1/70	Biolegend	1/200
CD11b-Pacific Blue	M1/70	Biolegend	1/200
CD11b-PE	M1/70	Biolegend	1/300
CD51-biotin	RMV-7	Biolegend	1/300
CD62E-PE	UZ6	Santa Cruz	1/20
CD62E	RME-1	Biolegend	10 μg mL^{-1}
Sav-BV785	N/A	BioLegend	1/300
Sav-BV605	N/A	Biolegend	1/300
FVS700	N/A	BD Biosciences	1/2500

Anti-human and mouse

**Antibodies**	**Clones**	**Suppliers**	**Dilution**
BrdU-PE	Bu20a	BioLegend	1/20
Ki67-AF700	B56	BD Pharmingen	1/20

IMMUNOBLOTTING

**Antibodies**	**Clones**	**Suppliers**	**Dilution**
Revert total transferred protein stain	N/A	Li-Cor	N/A
Phospho-Akt (Ser473)	D9E	Cell Signalling Technology	1/1000
Akt Total (pan)	C67E7	Cell Signalling Technology	1/1000
Phospho-NF-κb p65 (Ser536)	93H1	Cell Signalling Technology	1/1000
NF-κb p65 Total	C14E12	Cell Signalling Technology	1/1000
Phospho-p70 S6 Kinase (Ser371)	Polyclonal	Cell Signalling Technology	1/1000
Phospho-4E-BP1 (Thr37/46)	236B4	Cell Signalling Technology	1/1000
Beta-actin	AC-15	Novus Biologicals	1/5000
GAPDH	Polyclonal	Abcam	1/1000
Anti-Chicken IgG (H+L) (IRDye® 680RD)	N/A	Li-Cor	1/10000
Anti-Mouse IgG (H+L) (Dylight 680)	N/A	Cell Signalling Technology	1/10000
Anti-Rabbit IgG (H+L) (Dylight 800) (4X PEG Conjugate)	N/A	Cell Signalling Technology	1/10000
