# Supplementary material for: Endothelial E-selectin inhibition improves acute myeloid leukaemia therapy by disrupting vascular niche-mediated chemoresistance
Source: Nat Commun. 2020 Apr 27;11:2042. doi: 10.1038/s41467-020-15817-5 (PMC7184728; doi:10.1038/s41467-020-15817-5)
Supplement: Supplementary file 2 — Reporting summary [file 41467_2020_15817_MOESM2_ESM.pdf]

nature research

Corresponding author(s): Winkler I.G.

Last updated by author(s): Feb 21, 2020

Reporting Summary

Nature Research wishes to improve the reproducibility of the work that we publish. This form provides structure for consistency and transparency in reporting. For further information on Nature Research policies, see Authors & Referees and the Editorial Policy Checklist .

Statistics

For all statistical analyses, confirm that the following items are present in the figure legend, table legend, main text, or Methods section.

n/a	Confirmed	
☐	☒	The exact sample size (*n*) for each experimental group/condition, given as a discrete number and unit of measurement
☐	☒	A statement on whether measurements were taken from distinct samples or whether the same sample was measured repeatedly
☐	☒	The statistical test(s) used AND whether they are one- or two-sided<br>*Only common tests should be described solely by name; describe more complex techniques in the Methods section.*
☐	☒	A description of all covariates tested
☐	☒	A description of any assumptions or corrections, such as tests of normality and adjustment for multiple comparisons
☐	☒	A full description of the statistical parameters including central tendency (e.g. means) or other basic estimates (e.g. regression coefficient) AND variation (e.g. standard deviation) or associated estimates of uncertainty (e.g. confidence intervals)
☐	☒	For null hypothesis testing, the test statistic (e.g. *F*, *t*, *r*) with confidence intervals, effect sizes, degrees of freedom and *P* value noted<br>*Give P values as exact values whenever suitable.*
☒	☐	For Bayesian analysis, information on the choice of priors and Markov chain Monte Carlo settings
☒	☐	For hierarchical and complex designs, identification of the appropriate level for tests and full reporting of outcomes
☒	☐	Estimates of effect sizes (e.g. Cohen's *d*, Pearson's *r*), indicating how they were calculated

Our web collection on statistics for biologists contains articles on many of the points above.

Software and code

Policy information about availability of computer code

Data collection	Flow cytometry; BD FACS ARIA III Fusion, CytoFLEX S Beckman Coulter, CYAN ADP Beckman Coulter<br>Immunoblotting; LI-COR ; Beckman Coulter Ac.T DiFF.
Data analysis	Flow Collection software: Summit (Cyan), CytExpert 2.2.0.97 (CytoFlex), Diva 6 (Aria). Analysis software: All were imported as uncompensated files and re-compensated and analysed on FlowJo 7 (Cyan data) or FlowJo 10 (Cytoflex and Aria data). Excel 97-2003, Lcalc software (https://www.stemcell.com/l-calc-software.html), Image Studio Lite 5.2. (for LI-COR), GraphPad Prism v7

For manuscripts utilizing custom algorithms or software that are central to the research but not yet described in published literature, software must be made available to editors/reviewers. We strongly encourage code deposition in a community repository (e.g. GitHub). See the Nature Research guidelines for submitting code & software for further information.

Data

Policy information about availability of data

All manuscripts must include a data availability statement. This statement should provide the following information, where applicable:

- Accession codes, unique identifiers, or web links for publicly available datasets
- A list of figures that have associated raw data
- A description of any restrictions on data availability

All graphed datasets can be found in the Supplementary Source Data Files.

Field-specific reporting

Please select the one below that is the best fit for your research. If you are not sure, read the appropriate sections before making your selection.

Life sciences study design

All studies must disclose on these points even when the disclosure is negative.

Sample size	Sample sizes were determined based on the numbers used for previous experiments, which were sufficient to generate statistically significant results
Data exclusions	No data were excluded.
Replication	All experiments were replicated at least twice (as indicated in figure legends).<br>All attempts at replicating the observations described in this manuscript were successful.
Randomization	Recipient mice were randomly allocated to each group at the start of experiment. Mice were randomly assigned to the experimental groups for treatment with chemotherapy, GMI-1271 or vehicle
Blinding	Blinding was not performed for group allocations during experiments. Blinding was not needed for analysis because objective readouts were used in all experiments, and all samples were analyzed in the same way regardless of the groups

Reporting for specific materials, systems and methods

We require information from authors about some types of materials, experimental systems and methods used in many studies. Here, indicate whether each material, system or method listed is relevant to your study. If you are not sure if a list item applies to your research, read the appropriate section before selecting a response.

Materials & experimental systems

n/a	Involved in the study	
☐	☒	Antibodies
☐	☒	Eukaryotic cell lines
☒	☐	Palaeontology
☐	☒	Animals and other organisms
☐	☒	Human research participants
☒	☐	Clinical data

Methods

n/a	Involved in the study	
☒	☐	ChIP-seq
☐	☒	Flow cytometry
☒	☐	MRI-based neuroimaging

Antibodies

Antibodies used

FLOW CYTOMETRY

Anti-human
CD3-PacificBlue Cat # 300431 Biolegend clone UCHT1 lot B156239 (1:50)
CD19-PacificBlue Cat # 560353 Biolegend clone HIB19 lot 68917 (1:50)
CD33-BV421 Cat# 303416 BioLegend clone WM53 lot B165684 (1:40)
CD34-PE Cat# 343605 BioLegend clone 561 (1:10)
CD38-PECY7 Cat# 303516 BioLegend clone HIT2 lot B177096 (1:40)
IgM-AF647 Cat # 709-606-073 Jackson Immunoresearch
CD62E-PE Cat # 322605 Biolegend clone HCD62E lot B213130 (1:20)
CD62E-APC cat # 336012 Biolegend clone HAE-1f lot B194636 (1:20)
Ki67-A700 Cat # 561277 BD Pharmingen clone B56 lot 8264987 (1:21)

Anti-mouse
B220-PerCPCy5.5 Cat # 103236 BioLegend clone RA3-6B2 lot B209575 (1:250)
CD3- PerCPCy5.5 Cat # 100218 BioLegend clone 145-2C11 lot B249753 (1:250)
CD5- PerCPCy5.5 Cat # 100624 BioLegend clone 53-7.3 lot B278424 (1:250)
Ter119- PerCPCy5.5 Cat # 116228 BioLegend clone TER-119 lot B257070 (1:250)
Gr1- PerCPCy5.5 Cat # 108428 BioLegend clone RB6-8C5 lot B200811 (1:300)
Sca1-PECY7 Cat # 108114 BioLegend clone D7 lot B260282 (1:150)
Sca1-BV510 Cat # 108129 BioLegend clone D7 lot B262926 (1:100)
CD48-Pacific Blue Cat # 103418 BioLegend clone HM48-1 lot B218789 (1:300)
CD150-PE Cat # 115910 BioLegend clone TC15-12F12.2 lot B210719 (1:200)
Kit(CD117)-APCH7 Cat # 560250 BD Pharmingen clone 2B8 (1:200)
Kit(CD117)-PE Cat # 561075 BD Pharmingen clone 2B8 lot B131400 (1:200)

Kit(CD117)-APCCY7 Cat # 105826 BioLegend clone 2B8 lot B193535 (1:200)
Kit(CD117)-APC Cat # 105812 BioLegend clone 2B8 lot B199149 (1:200)
CD48-PerCPCy5.5 Cat # 103422 BioLegend clone HM48-1 lot B144102 (1:200)
CD45.1-PE Cat # 561872 BD Pharmingen clone A20 lot B155717 (1:300)
CD45.1-FITC Cat # 110706 Biolegend clone A20 lot B121038 (1:200)
CD45.2-APC Cat # 561875 BD Pharmingen clone 104 lot B213058 (1:300)
CD45-APCCY7 Cat # 103116 Biolegend clone 30F11 lot B257634 (1:300)
CD31-APC Cat # 102410 Biolegend clone 390 lot B144753 (1:200)
CD51-biotin Cat # 104104 Biolegend clone RMV-7 lot B167752 (1:300)
CD11b-PECY7 Cat # 101216 BioLegend clone M1/70 lot B203625 (1:200)
CD11b-Pacific Blue Cat # 101224 BioLegend clone M1/70 lot B247651 (1:200)
CD11b-PE Cat # 101208 BioLegend clone M1/70 lot B208137 (1:300)
CD62E-PE cat 59766 santa cruz clone UZ6 lot B0113 (1:20)
CD62E clone RME-1 cat # 148803 Biolegend lot B198645 (10 μg mL-1)
BrdU-PE Cat # 339812 Biolegend clone Bu20A lot B171614 (1:20)
Sav-BV785 Cat # 405249 Biolegend lot B237560 (1:300)
Sav-BV605 Cat # 405229 Biolegend lot B186807 (1:300)
FVS700 Cat # 564997 BD biosciences (1:2500)

IMMUNOBLOTTING

REVERT Total transferred protein stain Cat # 926-11015 Licor
Phospho-Akt (Ser473) clone D9E cat# 4060S Cell Signalling Technology lot 23 (1:1000)
Akt Total (pan) clone C67E7 cat#4691 Cell Signalling Technology (1:1000)
Phospho-NF-κb p65 (Ser536) clone 93H1 cat# 3033S Cell Signalling Technology lot 16 (1:1000)
NF-κB p65 (clone D14E12) XP® cat# 8242S Cell Signalling Technology lot 9 (1:1000)
Phospho-p70 S6 Kinase (Ser371) Polyclonal cat#9208 Cell Signalling Technology lot 4 (1:1000)
Phospho-4E-BP1 (Thr37/46) (clone 236B4) cat#2855 Cell Signalling Technology lot 23 (1:1000)
Beta-actin clone AC-15 cat# NOVNB600-501 Cell Signalling Technology lot 077M4757V (1:5000)
GAPDH Polyclonal cat#9485 Abcam (1:1000)
Anti-Chicken IgG (H+L) (IRDye® 680RD) cat# LCR-926-68075 Licor lot C70201-05 (1:10000)
Anti-Mouse IgG (H+L) (Dylight 680) cat#5470 Cell Signalling Technology lot 7 (1:10000)
Anti-Rabbit IgG (H+L) (Dylight 800 4X PEG Conjugate) cat# 5151P Cell Signalling Technology lot 9 (1:10000)

Validation

All antibodies were validated by manufacturers for the applications and species used in this study, and were supported by publications. See manufacturers websites
for validation statements (www.biolegend.com; www.bdbiosciences.com; www.cellsignal.com; https://www.abcam.com), found in technical data sheet.
All antibodies were tested by titrating it and matching isotype control against known reacting cell types in parallel.
All experiment included appropriate isotype control, and FMO (full stain minus one) controls.

Eukaryotic cell lines

Policy information about cell lines

Cell line source(s)

KG-1a (ATCC CCL-246.1), BMEC-1 (ATCC CRL-3421), RAW 264.7 (ATCC TIB-71) stably transfected with NF-κB response element from the SELE gene promotor driving GFP reporter expression (Stacey, K.J., et al. The molecular basis for the lack of immunostimulatory activity of vertebrate DNA. J Immunol 170, 3614-3620 (2003).

Authentication

Cell lines were purchased directly from ATCC. Apart from testing for mycoplasma contamination, no other authentication performed.

Mycoplasma contamination

All cells were tested negative for mycoplasma contamination

Commonly misidentified lines
(See ICLAC register)

None.

Animals and other organisms

Policy information about studies involving animals; ARRIVE guidelines recommended for reporting animal research

Laboratory animals

All mice were 8 to 16 weeks at start of experiment. Both male and female mice were used. Mice strains used were C57BL/6J and B6.SJL-Ptprc a

Wild animals

This study did not involve wild animals.

Field-collected samples	This study did not involve animals collected from the field.
Ethics oversight	All procedures involving animals were approved by Animal Ethics Committee of University of Queensland, Australia and Human ethics committees (Queensland Metro South HEC, and Peter MacCallum Cancer Centre HEC) as indicated in text (methods section page 17)

Note that full information on the approval of the study protocol must also be provided in the manuscript.

Human research participants

Policy information about studies involving human research participants

Population characteristics	Patients presenting with Myeloid Leukaemia and hip replacement patients.
Recruitment	Patients were invited to consent to an additional portion of blood or excess/discarded bone marrow being collected for research at the same time as standard of care treatment.
Ethics oversight	Collection of patient cells approved by Human Research Ethics Committee Queensland Metro South, Australia and PeterMac Human Ethics Committee, Australia

Note that full information on the approval of the study protocol must also be provided in the manuscript.

Flow Cytometry

Plots

Confirm that:

- [x] The axis labels state the marker and fluorochrome used (e.g. CD4-FITC).
- [x] The axis scales are clearly visible. Include numbers along axes only for bottom left plot of group (a 'group' is an analysis of identical markers).
- [x] All plots are contour plots with outliers or pseudocolor plots.
- [x] A numerical value for number of cells or percentage (with statistics) is provided.

Methodology

Sample preparation	Bone marrow and peripheral blood were prepared in single cell suspension in PBS with 2%serum. Bone marrow cells were then either used whole (total bone marrow) or were cKIT/CD117-enriched using MACS beads and AUTOMACS POSSEL purification as described by manufactorer (Miltenyi). Red blood cells were lysed using ammonium chloride solution (140mM) for 5 minutes at room temperature before wash. All cells were filtered through 40uM cell filters before flow cytometry or for FACS.
Instrument	Cyan ADP, Beckman Coulter; CytoFlex S, Beckman Coulter; BD FACS ARIA III Fusion
Software	Collection software: Summit (Cyan), CytExpert 2.2.0.97 (CytoFlex), Diva 6 (Aria). Analysis software: All were imported as uncompensated files and re-compensated and analysed on FlowJo 7 (Cyan data) or FlowJo 10 (Cytoflex and Aria data)
Cell population abundance	Cell population abundance: (1) Leukaemic bone marrow contained 20% to 95% GFP+ leukaemic blasts, (2) Leukaemic blood leukocytes contained 0.1% to 80% leukaemic blasts. CD117-MACS enriched bone marrow contained >95% blasts, CD34+ enriched patient cells contained >75% CD34+ cells.
Gating strategy	CTYOFLEX.<br>FSC-A v. SSC-A for mononuclear cells; pulse width v. FSC-A, 7AAD- for live cells; then (1) MURINE CELLS; Lineage cocktail v. KIT, KIT v. GFP, E-selectin-IgM-AF647 (2) HUMAN CELLS; Lineage cocktail vs. CD34, CD34 vs CD38, E-selectin-IgM-AF647

- [x] Tick this box to confirm that a figure exemplifying the gating strategy is provided in the Supplementary Information.
